# Peripheral blood T-lymphocyte subsets provide prediction of early postoperative recurrence in elderly patients with lung cancer: model development and external validation

**DOI:** 10.3389/fonc.2026.1948080

**Published:** 2026-09-07

**Authors:** Hongtai Xiong, Yuming Liu, Yanyuan Du, Peiyi Yu, Xudong Lei, Zhizheng Zhao, Honggang Zheng

**Affiliations:** 1 Department of Oncology, Guang’anmen Hospital, China Academy of Chinese Medical Sciences, Beijing, China; 2 Center of Integrated Traditional Chinese and Western Medicine Oncology, Sun Yat-sen University Cancer Center Gansu Hospital, Lanzhou, Gansu, China

**Keywords:** early recurrence, elderly patients with lung cancer, machine learning, predictive model, risk factors, T-lymphocyte subsets

## Abstract

**Background:**

With the accelerating aging of the population, the number of elderly patients with lung cancer continues to increase. Although surgery remains an important treatment modality for lung cancer, early recurrence (ER) is still a major factor affecting long-term survival in elderly patients.

**Purpose:**

This study aimed to integrate peripheral blood T-lymphocyte subset indicators with clinicopathological characteristics to develop and externally validate a machine learning-based prediction model for ER in elderly patients with lung cancer.

**Methods:**

This study was designed as a multicenter retrospective cohort study. ER was defined as local recurrence or distant metastasis within 2 years after surgery. A total of 23 candidate predictors were included, and dual feature selection was performed using the Boruta algorithm and LASSO regression, followed by a systematic comparison of 14 machine learning algorithms and 7 ensemble optimization strategies. Model performance was evaluated using the area under the receiver operating characteristic curve (AUROC), area under the precision-recall curve (AUPRC), calibration curve, Brier score, and decision curve analysis. SHAP was used to interpret the optimal model.

**Results:**

A total of 893 elderly patients with lung cancer were included in this study. After dual feature selection, five predictors were ultimately retained: tumor stage, total T-lymphocyte percentage, body mass index (BMI), Karnofsky performance scale (KPS), and suppressor/cytotoxic T-lymphocyte percentage. After ensemble optimization, SMOTE-Stacking achieved the best performance in internal validation. In external validation, among the four models belonging to the statistically equivalent top-performing tier, Soft Voting Ensemble (SV) demonstrated the most robust performance. SHAP analysis indicated that tumor stage and total T-lymphocyte percentage were the two core drivers of model prediction, both exhibiting clear nonlinear threshold effects.

**Conclusion:**

ER in elderly patients with lung cancer results from the combined effects of tumor burden, host functional status, and T-lymphocyte immune imbalance. A machine learning model integrating peripheral blood T-lymphocyte subsets has noninvasive and clinically translational potential and may provide evidence for ER risk stratification, individualized follow-up, and early intervention decision-making in elderly patients with lung cancer.

## 1 Introduction

Based on data released by the IARC, lung cancer accounted for 12.4% of new cancer cases worldwide in 2022, and its death toll represented 18.7% of all malignant tumor fatalities, with both incidence and mortality rates remaining persistently high (1, 2). In China, the population aged over 60 years constituted 18% in 2020 and is expected to increase to 39% by 2050 (3). With the aging of the population and the widespread use of high-resolution computed tomography (CT), the number of elderly patients with lung cancer (≥60 years) continues to increase. Globally, from 1992 to 2021, the number of incident lung cancer cases and deaths among individuals aged 60 years and older increased from 837,178 to 1,799,625 and from 819,480 to 1,628,656, respectively (4). The diagnosis and treatment of elderly patients with lung cancer have therefore become a major challenge for clinicians.

In recent years, advances in video-assisted thoracic surgery (VATS) have broadened both the indications and feasibility of thoracic surgical resection, making this approach applicable even in elderly patients (5). Shi et al. reported that surgical intervention significantly improved survival among patients aged ≥70 years or ≥80 years with early-stage non-small cell lung cancer (NSCLC) (6). Although VATS enables effective resection of tumor tissue with relatively limited surgical trauma, 30%–75% of patients with NSCLC still face postoperative recurrence (7). The risk of recurrence increases with disease stage, rising from 33.9% in stage IA to 37.8% in stage IB, 57.9% in stage IIB, and 62.8% in stage IIIA (8). Compared with younger patients, elderly patients with lung cancer are more likely to present with immunosenescence, chronic inflammatory status, and multiple comorbidities, which may place them at an even higher risk of postoperative recurrence and metastasis (9).

Early recurrence (ER) refers to local relapse or distant metastasis occurring within 2 years after surgery (10). Studies have demonstrated that ER is associated with pathological stage, tumor size, histological subtype, and vascular invasion (11, 12). Nevertheless, given the biological heterogeneity of lung cancer, reliance solely on clinicopathological parameters makes it difficult to accurately capture tumor biological behavior. In recent years, evaluation of recurrence risk in cancer has progressively extended beyond conventional pathological staging and clinical characteristics to include indicators reflecting host immune status (13, 14). Wang et al. found that immune microenvironmental features, including aberrant macrophage infiltration and memory B cells, were closely linked to ER in lung cancer (15). However, direct assessment of the intratumoral immune environment is hindered by several clinical drawbacks, including its invasive nature, the time required, and the impracticality of repeated sampling, making this approach particularly difficult in patients with lung cancer (16). T-lymphocytes, a major immune cell population in peripheral blood, are integral components of both innate and adaptive immunity and play critical roles in antitumor responses (17). Within the T-lymphocyte subsets, CD3+ T cells indicate overall immune competence, CD3+CD4+ T cells coordinate immune responses, CD3+CD8+ T cells mediate recognition and cytotoxic killing of tumor cells, whereas regulatory T cells (Treg) are involved in immune tolerance (18). Recent studies have shown that peripheral blood T-lymphocyte subsets are associated with the overall immune status of patients and may serve as noninvasive tools for predicting responses to immunotherapy and EGFR-TKI treatment in patients with lung cancer (19, 20), as well as for predicting survival (21, 22). However, few studies have investigated the potential value of T-lymphocyte subsets in predicting ER in lung cancer.

Machine learning techniques, by virtue of their advantages in handling high-dimensional data, modeling nonlinear relationships, and constructing predictive models, have created new opportunities for developing individualized risk prediction tools (23). First, recursive feature elimination or tree-based feature selection can automatically identify the optimal subset from candidate predictors, thereby reducing redundancy. Second, ensemble learning strategies combine multiple learners to substantially improve predictive robustness and generalizability while mitigating the overfitting risk inherent to any single learner. In addition, the development of Shapley additive explanations (SHAP) has enabled visualization of the internal decision logic of models, thereby enhancing clinicians’ confidence in and acceptance of these approaches. Compared with traditional logistic regression (LR), machine learning models can integrate multicategory immune parameters and clinicopathological features, allowing more effective identification of subgroups at high risk of recurrence (24).

Based on this, the study aims to use machine learning algorithms to integrate peripheral blood T-lymphocyte subset data and clinicopathological characteristics in elderly patients with lung cancer, in order to construct and externally validate a prediction model for ER. The goal is to establish a risk assessment tool with good discrimination, calibration, and clinical utility, thereby providing a basis for postoperative follow-up management, early intervention decision-making, and precision treatment in elderly patients with lung cancer.

## 2 Methods

### 2.1 Study design

This multicenter retrospective cohort study aimed to develop and validate a machine learning-based model for predicting ER in elderly patients with lung cancer. Data for the development cohort were obtained from Guang’anmen Hospital, China Academy of Chinese Medical Sciences, whereas data for the external validation cohort were obtained from Sun Yat-sen University Cancer Center Gansu Hospital. The enrollment period for both cohorts extended from January 1, 2019, to January 1, 2024. The inclusion criteria were as follows: (1) age of 60 years or older; (2) NSCLC confirmed by histopathological examination; (3) tumor stage I–IIIA, with no known distant metastasis at surgery; (4) curative-intent VATS with R0 resection and standard lymph node dissection; (5) analyzable follow-up data, including the earliest confirmed date of recurrence or metastasis, or completion of 2 years of postoperative follow-up without an outcome event; (6) overall survival longer than 2 years. Patients were excluded if any of the following conditions were present: (1) hematopoietic dysfunction, severe infection, or immune system disorders; (2) preoperative neoadjuvant radiotherapy, chemotherapy, or immunotherapy; (3) prior lobectomy, segmentectomy, or unilateral pneumonectomy; (4) concurrent other malignancies; (5) Karnofsky performance scale (KPS) <60. The study was designed and reported in accordance with the TRIPOD+AI statement (25), and risk of bias was assessed with reference to PROBAST+AI (26). This study complied with the principles of the Declaration of Helsinki, as revised in 2024 (27). Ethics approval was granted by the ethics committees of Guang’anmen Hospital, China Academy of Chinese Medical Sciences (ethics approval No. 2024-191-KY), and Sun Yat-sen University Cancer Center Gansu Hospital (ethics approval No. A202601070004).

### 2.2 Definition of outcome events

ER was defined as local recurrence or distant metastasis within 2 years after surgery (28). Local recurrence referred to a newly developed lesion at the resection margin, bronchial stump, ipsilateral hilar or mediastinal lymph nodes, which was confirmed to be malignant by imaging modalities such as CT, magnetic resonance imaging (MRI), or PET-CT, or by histopathological biopsy (29). Distant metastasis was defined as a newly detected metastatic lesion in distant organs, including the brain, bone, liver, adrenal gland, or contralateral lung, diagnosed using CT, MRI, PET-CT, or other imaging examinations, with pathological confirmation by needle biopsy when required (30). All recurrence or metastasis events were independently reviewed and confirmed by at least two radiologists or clinicians. If a new pulmonary lesion was identified, a second primary lung cancer was excluded based on the Martini–Melamed criteria or subsequent pathological results (31). This study employed a fixed 2-year postoperative follow-up period. All patients meeting the inclusion criteria had complete 2-year outcome ascertainment. Consequently, no right censoring occurred within the prediction window, and all outcome labels were derived from fully observed data.

### 2.3 Candidate predictor selection

Based on clinical experience and previous literature, the following 23 candidate predictors were included: (1) demographic characteristics: age, sex, body mass index (BMI), and body surface area (BSA); (2) functional status: KPS; (3) lifestyle factor: smoking index; (4) comorbidities: hypertension, diabetes, coronary heart disease (CHD), chronic obstructive pulmonary disease (COPD), and family history of cancer; (5) peripheral blood T-lymphocyte subset indicators: total T-lymphocyte (CD3+) percentage, helper/inducer T-lymphocyte (Th, CD3+CD4+) percentage, suppressor/cytotoxic T-lymphocyte (Ts/Tc, CD3+CD8+) percentage, CD4/CD8 ratio, and Treg (CD3+CD4+CD25+CD127−) percentage; (6) postoperative complications: pneumonia, pleural effusion, and pulmonary atrophy; (7) tumor characteristics: tumor site, TNM stage, and pathological type; (8) surgical information: surgical modality. Demographic characteristics, KPS, smoking index, comorbidity information, and peripheral blood T-lymphocyte subset parameters were collected preoperatively, whereas postoperative complications, tumor characteristics, and surgical information were collected within 2 weeks after surgery. T-lymphocyte subsets were measured by flow cytometry (FCM) in accordance with the standard operating procedures of the clinical laboratories. Routine instrument calibration and internal quality control were performed at both centers, and T-lymphocyte subsets were identified using predefined gating criteria (detailed FCM procedures and gating strategies are provided in **Supplementary Material 2**). The T-lymphocyte subset variables used for model development were primarily recorded as relative proportions.

### 2.4 Sample size adequacy assessment

This study used a retrospective cohort design, and the sample size was determined by the clinical data actually available at each center. According to the minimum sample size framework for prediction models proposed by Riley et al. (32, 33), a *post-hoc* test of sample size adequacy was performed for the development cohort (N = 670; number of events = 136; event rate = 20.3%; number of candidate predictors, p = 23). Four criteria were applied: global shrinkage factor (S ≥ 0.9), the difference between apparent and adjusted Cox–Snell R² (≤ 0.05), precision of overall event rate estimation (δ ≤ 0.05), and precision of intercept estimation. For the external validation cohort (N = 223; events = 38; event rate = 17.0%), the corresponding external validation framework was applied (34), and the standard error of the C statistic was estimated using the Hanley–McNeil method.

### 2.5 Missing data processing

Missing values were imputed using multiple imputation by chained equations (MICE) (35), which iteratively models the conditional distribution of each incomplete variable given the others to generate plausible imputed values. The maximum number of iterations was set to 50, the median was used for initial imputation, and the random seed was set to 42 to ensure reproducibility. Variables with >50% missing values were excluded prior to imputation. Importantly, the imputation model was fitted on the training set only, and the validation sets were imputed using parameters derived from the training set to prevent data leakage (36).

### 2.6 Data partitioning strategy

The development cohort was randomly divided into training and internal validation sets in an 8:2 ratio using stratified sampling. Specifically, 80% of the cohort was used for model development, while the remaining 20% was used to evaluate model performance in internal validation. Stratified sampling was applied to maintain a comparable proportion of recurrence events between the training and validation sets. The random seed was set to 42 to ensure reproducible results. The external validation cohort served as an independent temporal and geographic dataset and was not involved in any stage of model training or tuning.

### 2.7 Feature selection and intersection fusion strategy

High-dimensional clinical data inevitably contain redundant or noisy variables and including all variables without selection not only increases the risk of overfitting but also compromises model interpretability and clinical utility (37). Therefore, this study adopted a dual feature selection strategy combining the Boruta algorithm and least absolute shrinkage and selection operator (LASSO) regression to identify outcome-associated predictors from two complementary methodological perspectives, using the intersection of the two methods as the final feature set for modeling. Feature selection was performed exclusively within the training set, and the validation sets were held out at every stage to prevent data leakage (38). The Boruta algorithm was implemented with an RF model (500 trees, maximum depth = 5, max_features = 0.5). Across 500 iterations, it compared the importance of original features against that of randomly permuted shadow features via statistical testing (α = 0.05), retaining all variables whose importance was significantly greater than that of the shadow features (39). LASSO was implemented using L1-regularized LR, with the optimal regularization parameter λ determined by 10-fold cross-validation using AUROC as the optimization objective; unimportant variables were shrunk to exactly zero, thereby achieving embedded variable selection (40). The two methods are complementary in principle: Boruta, as a nonparametric RF-based method, is well suited to capturing nonlinear effects and interactions, whereas LASSO, as a parametric penalized regression method, focuses on linear main effects and inherently handles collinearity (37). Using the intersection of features identified by both methods minimizes the probability of selecting false-positive predictors and retains only those validated across both methodological frameworks.

### 2.8 Machine learning algorithms and model ensemble optimization strategy

This study systematically evaluated the predictive performance of 14 machine learning algorithms, covering four major methodological categories: (1) Linear and generalized linear models: LR and generalized linear model with a binomial distribution (GLM-B), which served as the baseline linear classification framework. (2) Instance-based and kernel-based models: K-nearest neighbors (KNN) and support vector machine (SVM), which performed nonlinear classification via distance metrics and kernel functions, respectively. (3) Neural network model: multilayer perceptron (MLP), which captured complex feature interactions through multiple layers of nonlinear transformations. (4) Decision tree (DT) and ensemble learning models: DT and shallow decision tree (SDT) were used as base learners, and bagging-based models, including random forest (RF) and extremely randomized tree (ET), as well as boosting-based models, including adaptive boosting (AdaBoost), gradient boosting decision tree (GBDT), extreme gradient boosting (XGBoost), light gradient boosting machine (LightGBM), and categorical boosting (CatBoost), were further constructed. All models were encapsulated within a unified preprocessing–modeling pipeline, sequentially comprising MICE, StandardScaler, and the classifier, to prevent leakage of preprocessing parameters between the training and validation sets. Model selection and hyperparameter tuning were performed using a nested cross-validation strategy (5-fold stratified outer loop with 3-fold stratified RandomizedSearch in the inner loop, n_iter = 20), and 5-fold stratified out-of-fold (OOF) predicted probabilities from the training set served as the baseline for robust assessment of model generalizability. To address class imbalance between samples with and without recurrence, class-weight adjustment strategies tailored to each algorithm’s optimization mechanism were applied during training: LR, SVM, ET, and DT were configured with class_weight=‘balanced’; RF was configured with class_weight=‘balanced_subsample’ so that class weights were recalculated within the bootstrap sample of each tree; XGBoost and LightGBM up-weighted positive-sample gradients via the scale_pos_weight parameter; and CatBoost was configured with auto_class_weights=‘Balanced’. AdaBoost, GBDT, and MLP relied on the intrinsic amplification of minority-class residuals during boosting and gradient-based optimization, whereas GLM-B served as an unbalanced baseline comparator. To balance sensitivity and specificity, the classification threshold for each model was determined by maximizing Youden’s J index (J = Sensitivity + Specificity − 1) (41). For the 14 base models, thresholds were derived from the predicted probabilities on the internal validation set.

In class-imbalanced clinical prediction scenarios, a single classifier often has limited ability to identify minority-class cases (i.e., patients with recurrence or metastasis), resulting in insufficient sensitivity and low positive predictive value (42). To address this limitation, after identifying the optimal single base learner (highest AUROC on the internal validation set) and the Top-3 ranked models among the 14 candidates, this study systematically implemented seven ensemble optimization strategies, as detailed below. (1) BorderlineSMOTE. The BorderlineSMOTE algorithm (k = 5 nearest neighbors) was used to synthesize minority-class samples specifically located near the decision boundary, thereby alleviating class imbalance while limiting noise introduction. The optimal single base learner was then retrained, and the classification threshold maximizing the F1 score was determined by grid search (threshold range, 0.10–0.90; step size, 0.01). (2) Polynomial feature interaction (PolyFI). PolynomialFeatures (interaction_only=True) was used to generate all pairwise interaction terms among the original features, excluding higher-order power terms, to explicitly capture potential nonlinear synergistic effects between variables. (3) Stacking. The Top-3 models ranked by AUROC served as base learners, their OOF predicted probabilities generated through 5-fold cross-validation were used as meta-features, and an L2-regularized LR model served as the meta-learner. To reduce overfitting, the meta-learner did not receive the original features (passthrough = False) (43). (4) Soft voting ensemble (SV). The predicted probabilities output by the Top-3 base learners were averaged (arithmetic mean) to integrate model confidence at the probability level and generate the final prediction. (5) Weighted averaging ensemble (WAE). The ratio of each base learner’s AUROC on the internal validation set to the sum of AUROCs across all base learners was used as the weighting coefficient, achieving performance-weighted fusion of predicted probabilities. (6) SMOTE-Stacking. Standard SMOTE oversampling was first applied to the training set to balance the class distribution; a stacking ensemble was then constructed on the oversampled data, simultaneously addressing class imbalance and single-model performance limitations. (7) Bayesian hyperparameter optimization (BO-TPE). With the TPE sampler implemented in the Optuna framework, 100 Bayesian optimization trials were performed for the optimal single base learner. The search space included two core hyperparameters, n_estimators (50–500) and learning_rate (0.01–1.0), and the mean area under the precision-recall curve (AUPRC) obtained by 5-fold cross-validation served as the optimization objective. Of these seven strategies, only BorderlineSMOTE used grid search to identify the F1-maximizing threshold; the remaining six used classification thresholds derived from 5-fold stratified OOF predicted probabilities on the training set, to avoid optimistic bias from repeated comparisons on the internal validation set during the ensemble stage.

### 2.9 Model performance evaluation and optimal model selection strategy

To comprehensively evaluate the statistical performance and clinical utility of the models, this study conducted a systematic assessment from three complementary dimensions: discrimination, calibration, and clinical net benefit. (1) Discrimination assessment: The primary evaluation metric was AUROC. Secondary metrics included AUPRC, sensitivity, specificity, F1 score, positive predictive value (PPV), and negative predictive value (NPV). AUPRC provides a more comprehensive evaluation for class-imbalanced datasets (44). (2) Calibration assessment: Calibration curves and the Brier score were used to evaluate the agreement between predicted and observed probabilities of recurrence (45); log loss was additionally reported to capture deviations in the predicted probability distribution. The Brier score ranges from 0 to 1, with values closer to 0 indicating better calibration. (3) Clinical utility assessment: Decision curve analysis (DCA) was used to evaluate the net benefit of each model across a range of threshold probabilities relative to the “treat-all” and “treat-none” reference strategies (46). Pairwise AUROC comparisons across models were performed using the DeLong test, with *p* < 0.05 considered statistically significant (47).

The optimal model was determined using a three-stage evaluation strategy. In the first stage, among the 14 base models, the highest-AUROC learner on the internal validation set was identified for the three single-base-learner strategies (BorderlineSMOTE, PolyFI, and BO-TPE), and the Top-3 base learners by internal validation AUROC were identified for the four Top-3-based probability-fusion strategies (Stacking, SV, WAE, and SMOTE-Stacking). In the second stage, among the seven ensemble optimization strategies, the highest AUROC on the internal validation set was prespecified as the primary criterion for selecting the optimal fusion strategy, with AUPRC, Brier score, and DCA net benefit as secondary metrics. The external validation cohort served exclusively as an independent assessment of generalizability and was not involved in the first- or second-stage screening. Notably, in the WAE strategy, the weighting coefficients were determined using AUROC values from the internal validation set; once established, these weights were fixed and were not updated using external data during external validation or deployment. Thus, internal validation information was used only for determining fusion weights, while downstream model selection and external generalizability assessment remained independent. In the third stage, for candidate fusion models belonging to the “statistically equivalent top-performing tier” in the internal validation screening (defined as an AUROC difference ≤ 0.005 and a paired DeLong test *p* > 0.05), the final model for clinical deployment was confirmed based on overall robustness in the external validation cohort, including external AUROC, Brier score, DCA net benefit, and the extent of cross-cohort performance attenuation. This final model was also used for subsequent SHAP interpretability analysis. The 95% confidence intervals for AUROC were estimated using 1000 stratified bootstrap resamples. All other secondary metrics (AUPRC, sensitivity, specificity, PPV, NPV, F1 score, Brier score, and log loss) were reported as point estimates.

### 2.10 External validation of ensemble models

The seven ensemble models and the Top-3 base models were directly applied to the external validation cohort from Sun Yat-sen University Cancer Center Gansu Hospital. During external validation, the preprocessing parameters derived from the development cohort, including standardization parameters and missing data imputation parameters, were strictly used, without any retraining or parameter adjustment. The evaluation metrics were consistent with those used in internal validation.

### 2.11 Model interpretability analysis

SHAP was used to interpret the optimal model (48, 49). SHAP is grounded in Shapley values from cooperative game theory and quantifies the contribution of each feature to an individual prediction. In this study, the following SHAP visualizations were generated: (1) a bar plot showing global feature importance ranked by mean absolute SHAP value; (2) a beeswarm plot showing the distribution of SHAP values across samples and their relationships with feature values; (3) dependence plots revealing the nonlinear relationships between key features and model outputs; (4) waterfall plots illustrating the stepwise contributions of individual features in representative correctly classified cases; and (5) a force plot visualizing each feature’s contribution for an individual patient as color-coded arrows (red for contributions that increase the predicted probability and blue for those that decrease it), illustrating the cumulative process by which the prediction moves from the baseline value E[f(X)] to f(x).

### 2.12 Statistical analysis

Continuous variables were expressed as median with interquartile range [M (Q1, Q3)], and between-group comparisons were performed using the Mann–Whitney U test or Kruskal–Wallis test. Categorical variables were presented as frequencies and percentages [n (%)], and between-group comparisons were performed using the *χ²* test. All machine learning modeling and analyses were conducted in Python 3.10. Statistical significance was defined as *p* < 0.05.

## 3 Results

### 3.1 Baseline characteristics of the study cohort

**Figure 1:**
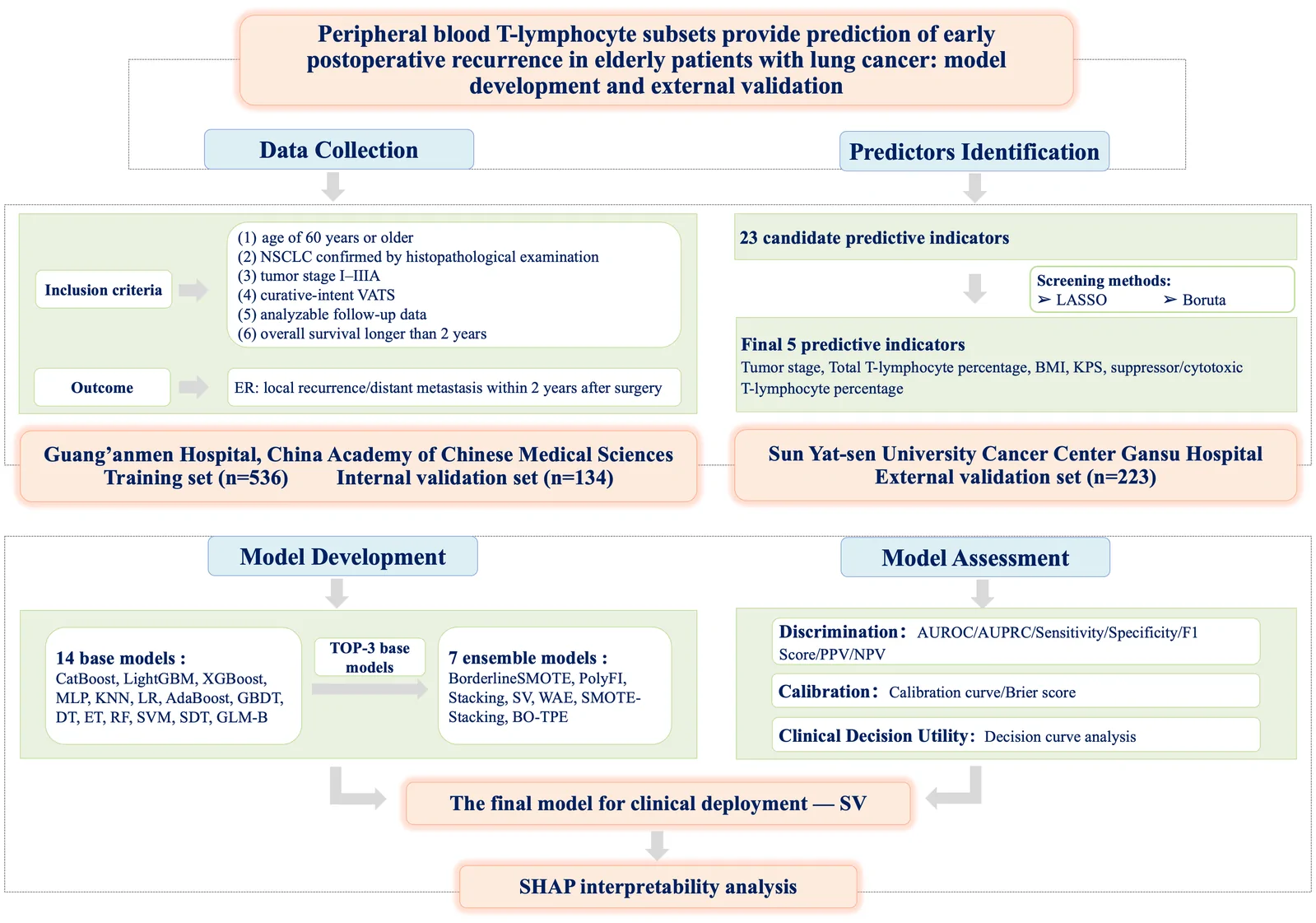
Study design and workflow.

The overall study workflow is shown in **Figure 1**. A total of 893 elderly patients with lung cancer after surgery were included in the final analysis, including 670 patients in the development cohort and 223 patients in the external validation cohort. In the development cohort, 136 patients (20.30%) developed recurrence or metastasis within 2 years, while 534 patients (79.70%) did not experience recurrence. In the external validation cohort, ER occurred in 38 patients (17.0%), whereas 185 patients (83.0%) remained recurrence-free. The development cohort was randomly split in a stratified manner at a ratio of 8:2 into a training set (n = 536, with 109 recurrence events [20.3%]) and an internal validation set (n = 134, with 27 recurrence events [20.1%]). The baseline characteristics of these three groups are shown in **Table 1**.

**Table 1:** Baseline characteristics of the training set, internal validation set, and external validation set.

Variable	Training set (n=536)	Internal validation set (n=134)	External validation set (n=223)	*p*
Age	67.00 (64.00-71.00)	67.00 (64.00-72.00)	67.00 (65.00-69.00)	0.094
Sex				<0.001
Male	277 (51.7%)	62 (46.3%)	154 (69.1%)	
Female	259 (48.3%)	72 (53.7%)	69 (30.9%)	
BMI	23.62 (21.56-26.12)	24.03 (21.84-26.19)	23.18 (21.23-24.78)	0.044
BSA	1.68 (1.56-1.80)	1.68 (1.57-1.79)	1.75 (1.63-1.84)	<0.001
KPS	90.00 (80.00-90.00)	90.00 (80.00-90.00)	80.00 (80.00-90.00)	<0.001
Smoking Index	0.00 (0.00-600.00)	0.00 (0.00-600.00)	0.00 (0.00-400.00)	0.594
Hypertension				<0.001
No	299 (55.8%)	81 (60.4%)	158 (76.3%)	
Yes	237 (44.2%)	53 (39.6%)	49 (23.7%)	
Diabetes				0.030
No	412 (76.9%)	109 (81.3%)	176 (85.4%)	
Yes	124 (23.1%)	25 (18.7%)	30 (14.6%)	
CHD				<0.001
No	440 (82.1%)	111 (82.8%)	196 (95.1%)	
Yes	96 (17.9%)	23 (17.2%)	10 (4.9%)	
COPD				0.030
No	489 (91.2%)	128 (95.5%)	198 (96.1%)	
Yes	47 (8.8%)	6 (4.5%)	8 (3.9%)	
Family history of cancer				<0.001
No	397 (74.1%)	97 (72.4%)	192 (93.2%)	
Yes	139 (25.9%)	37 (27.6%)	14 (6.8%)	
Total T (CD3+)	71.12 (60.24-77.91)	70.89 (59.37-77.08)	70.72 (59.13-77.14)	0.777
Th (CD3+CD4+)	40.99 (30.27-48.48)	40.98 (31.78-46.98)	42.41 (30.65-48.50)	0.813
Ts/Tc (CD3+CD8+)	28.47 (22.52-34.46)	30.05 (23.84-37.38)	25.94 (21.34-32.04)	<0.001
CD4/CD8	1.33 (0.97-1.80)	1.30 (0.92-1.79)	1.51 (1.00-1.92)	0.063
Treg (CD3+CD4+CD25+CD127-)	10.16 (8.18-12.28)	9.91 (8.29-12.30)	10.24 (7.88-12.12)	0.830
Pneumonia				<0.001
No	367 (68.7%)	105 (78.4%)	193 (89.8%)	
Yes	167 (31.3%)	29 (21.6%)	22 (10.2%)	
Pleural effusion				0.037
No	421 (78.8%)	109 (81.3%)	153 (71.2%)	
Yes	113 (21.2%)	25 (18.7%)	62 (28.8%)	
Pulmonary atrophy				0.004
No	466 (87.3%)	117 (87.3%)	205 (95.3%)	
Yes	68 (12.7%)	17 (12.7%)	10 (4.7%)	
Tumor site				0.131
Left upper lobe	97 (18.2%)	23 (17.2%)	32 (14.3%)	
Left lower lobe	97 (18.2%)	30 (22.4%)	33 (14.8%)	
Right upper lobe	111 (20.8%)	29 (21.6%)	52 (23.3%)	
Right middle lobe	92 (17.3%)	27 (20.1%)	57 (25.6%)	
Right lower lobe	136 (25.5%)	25 (18.7%)	49 (22.0%)	
Stage				0.232
I	230 (42.9%)	51 (38.1%)	79 (35.4%)	
II	105 (19.6%)	34 (25.4%)	48 (21.5%)	
IIIA	201 (37.5%)	49 (36.6%)	96 (43.0%)	
Pathological type				<0.001
Adenocarcinoma	386 (72.0%)	93 (69.4%)	122 (54.7%)	
Squamous	100 (18.7%)	27 (20.1%)	70 (31.4%)	
Adenosquamous	20 (3.7%)	5 (3.7%)	8 (3.6%)	
Other	30 (5.6%)	9 (6.7%)	23 (10.3%)	
Surgical modality				0.083
Lobectomy	424 (81.2%)	101 (78.3%)	174 (81.3%)	
Segmentectomy	82 (15.7%)	23 (17.8%)	25 (11.7%)	
Wedge resection	16 (3.1%)	5 (3.9%)	15 (7.0%)	
ER				0.567
No	427 (79.7%)	107 (79.9%)	185 (83.0%)	
Yes	109 (20.3%)	27 (20.1%)	38 (17.0%)	

### 3.2 Results of dual feature selection

**Figure 2:**
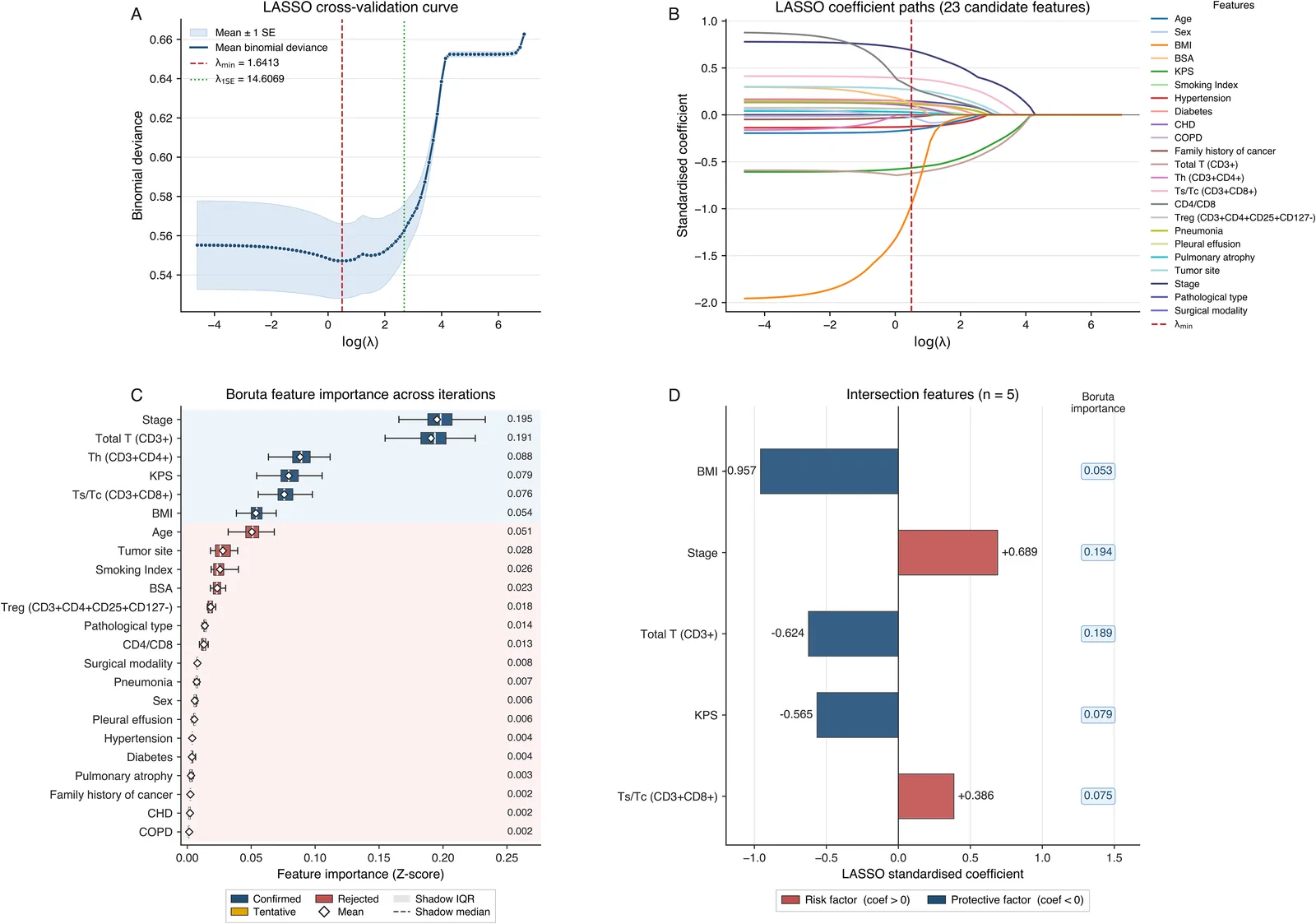
Dual feature selection based on LASSO and Boruta. **(A)** Ten-fold cross-validation curve for LASSO (L1-regularized logistic regression); **(B)** LASSO coefficient regularization path plot; **(C)** Boxplot of feature importance generated by Boruta; **(D)** Visualization of the direction and effect size of the five intersecting features (n = 5) jointly selected by LASSO and Boruta.

Using 10-fold cross-validation, LASSO regression identified the optimal regularization parameter as λ = 1.641 (corresponding to C = 1/λ = 0.609). At this level of penalization, 20 candidate variables had nonzero coefficients and were retained in the LASSO-selected feature set (**Figures 2A, B**). After 500 iterations, the Boruta algorithm identified 6 features as statistically significant important variables out of the 23 candidate variables, while 17 variables were rejected and no tentative variables remained. Among these, 1 Boruta-confirmed variable was not selected by LASSO. The final intersection therefore yielded 5 core predictors for model development (**Figure 2C**): tumor stage, total T-lymphocyte percentage, BMI, KPS, and Ts/Tc percentage (**Figure 2D**). Among these, 2 predictors were lymphocyte-related indicators, underscoring the important value of the immune microenvironment in predicting postoperative recurrence; the remaining 3 predictors captured tumor stage, patient functional status, and nutritional status, together reflecting a three-dimensional recurrence-risk profile integrating tumor burden, host immunity, and overall systemic condition. Further analysis of the directions of the standardized LASSO coefficients showed that tumor stage (β = +0.689) and Ts/Tc percentage (β = +0.386) were independent risk factors, whereas BMI (β = −0.957), total T-lymphocyte percentage (β = −0.624), and KPS (β = −0.565) were protective factors (**Figure 2D**).

### 3.3 Results of sample size adequacy assessment

A total of 670 patients were included in the development cohort of this study, with 136 events. For the 23 candidate predictors, the Events Per Variable (EPV) was 5.9, which was below the conventional empirical threshold for model development (EPV ≥ 10) (50), suggesting that the initial candidate predictor set was relatively large in relation to the sample size. To reduce the risk of overfitting, and following the recommendations of Riley et al. (32, 33), we performed data-driven dimensionality reduction using a dual feature selection strategy based on Boruta and LASSO, ultimately retaining 5 predictors in the final model. This increased the EPV to 27.2 in the development cohort, while the training set (N = 536, 109 events) had an EPV of 21.8. Both values exceeded the robust modeling threshold recommended by Peduzzi et al. (50), indicating that the sample size was adequate for prediction model development. According to the minimum sample size framework for prediction models proposed by Riley et al. (32, 33), a *post hoc* assessment of sample size adequacy was conducted using an event rate of 20.3%, 5 final model predictors, and an anticipated Cox–Snell R² of 0.15. Four criteria were applied: global shrinkage factor (S ≥ 0.9), the difference between apparent and adjusted Cox–Snell R² (≤ 0.05), precision of overall event rate estimation (δ ≤ 0.05), and precision of intercept estimation. The calculated minimum required sample size was 275, with a minimum of 56 events. The development cohort in this study (N = 670; 136 events) satisfied all of these criteria. The external validation cohort (N = 223; 38 events; event rate, 17.0%) was evaluated according to the external validation framework of Riley et al. (34). Based on an anticipated C statistic of 0.75 and a target 95% CI half-width of 0.10, the Hanley–McNeil method estimated a minimum required sample size of 200 and a minimum of 34 events. The sample size of the external validation cohort in this study met the required criteria.

### 3.4 Comparison of base models

**Figure 3:**
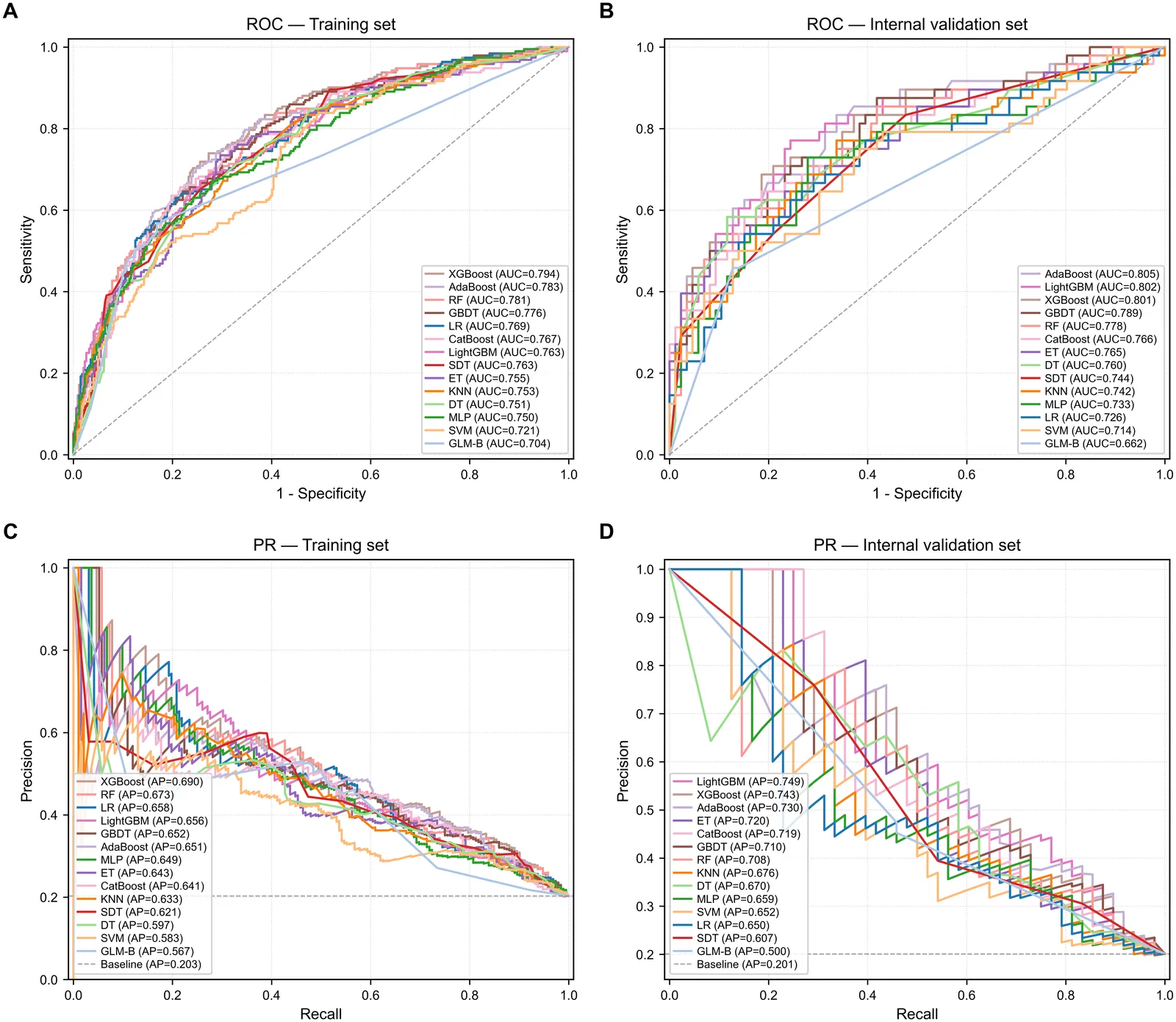
ROC and PR curves of the 14 basic models on the training set and internal validation set. **(A)** ROC curves for the training set; **(B)** ROC curves for the internal validation set; **(C)** PR curves for the training set; **(D)** PR curves for the internal validation set.

**Figure 4:**
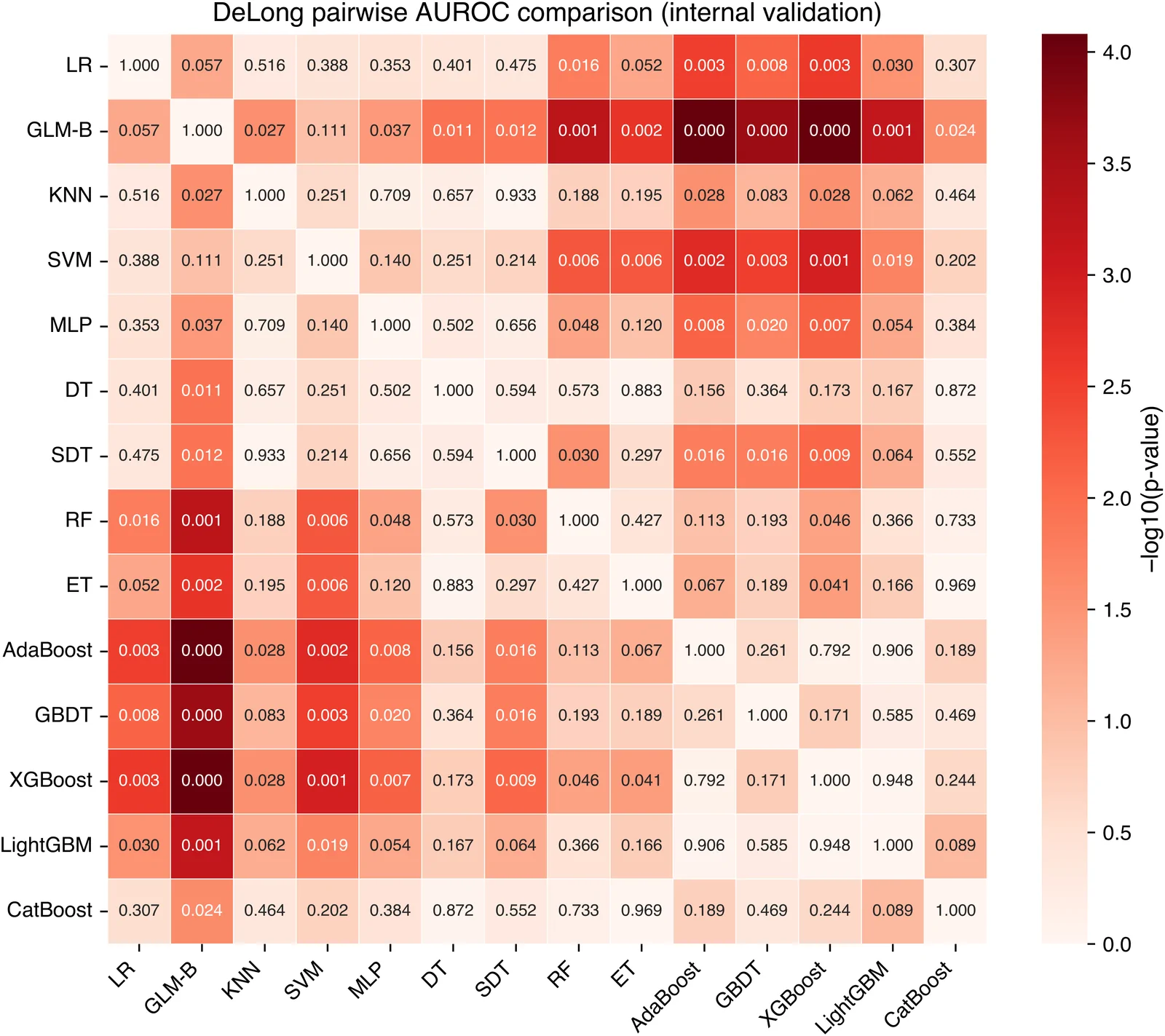
Heatmap of pairwise DeLong comparisons of AUROC among the 14 machine learning models in the internal validation set.

The performance of the 14 machine learning algorithms in the training set and internal validation set is summarized in **Table 2**, and the ROC and PR curves are shown in **Figure 3**. In terms of discrimination, AdaBoost ranked first in AUROC, achieving an AUROC of 0.805 (95% CI: 0.717–0.879) and an AUPRC of 0.730. LightGBM (AUROC = 0.802, 95% CI: 0.715–0.883; AUPRC = 0.749) and XGBoost (AUROC = 0.801, 95% CI: 0.717–0.876; AUPRC = 0.743) followed closely. The differences in AUROC among these three models were all within 0.005, their 95% CI overlapped substantially, and pairwise DeLong tests showed no statistically significant differences (*p* > 0.05) (**Figure 4**). GBDT (AUROC = 0.789, 95% CI: 0.701–0.866) and RF (AUROC = 0.778, 95% CI: 0.687–0.854) ranked next. All five belonged to ensemble tree-based models. Overall, boosting-based models (AdaBoost, LightGBM, XGBoost, and GBDT) showed better discrimination than bagging-based models (RF and ET) and non-ensemble algorithms. The discrimination of linear models (LR, AUROC = 0.726; GLM-B, AUROC = 0.662) and the shallow neural network model (MLP, AUROC = 0.733) was relatively limited, suggesting that linear assumptions were insufficient to fully characterize the nonlinear interactions among BMI, KPS, Total T, Ts/Tc, and stage in this dataset, while MLP failed to demonstrate the advantages of deep models under the constraints of a low-dimensional five-feature space and limited sample size. SVM (AUROC = 0.714) and SDT (AUROC = 0.744) showed intermediate performance. Although the single DT model (AUROC = 0.760) outperformed the linear baselines, its log loss (1.154) was substantially higher than that of the other models, indicating unstable probability estimation and further underscoring the importance of ensemble strategies in improving the robustness of weak learners.

**Table 2:** Performance comparison of the 14 base models in the training set and internal validation set.

Model	AUROC (95% CI)	AUPRC	Accuracy	Sensitivity	Specificity	PPV	NPV	F1	Brier	log loss
Training set										
CatBoost	0.767 (0.727-0.806)	0.641	0.700	0.766	0.663	0.559	0.835	0.646	0.191	0.579
LightGBM	0.763 (0.722-0.803)	0.656	0.670	0.740	0.631	0.528	0.813	0.616	0.195	0.595
XGBoost	0.794 (0.758-0.831)	0.690	0.733	0.714	0.744	0.609	0.823	0.657	0.181	0.542
MLP	0.750 (0.709-0.793)	0.649	0.664	0.698	0.645	0.523	0.793	0.598	0.187	0.555
KNN	0.753 (0.711-0.794)	0.633	0.638	0.833	0.529	0.497	0.850	0.623	0.187	0.580
LR	0.769 (0.727-0.811)	0.658	0.744	0.531	0.863	0.685	0.767	0.598	0.198	0.580
AdaBoost	0.783 (0.744-0.820)	0.651	0.718	0.750	0.701	0.583	0.834	0.656	0.199	0.585
GBDT	0.776 (0.738-0.815)	0.652	0.685	0.755	0.645	0.543	0.825	0.632	0.181	0.539
DT	0.751 (0.711-0.792)	0.597	0.709	0.484	0.834	0.620	0.744	0.544	0.208	0.878
ET	0.755 (0.716-0.799)	0.643	0.694	0.479	0.814	0.590	0.737	0.529	0.203	0.595
RF	0.781 (0.740-0.820)	0.673	0.636	0.849	0.517	0.495	0.860	0.626	0.185	0.551
SVM	0.721 (0.680-0.766)	0.583	0.554	0.911	0.355	0.441	0.878	0.594	0.196	0.576
SDT	0.763 (0.723-0.802)	0.621	0.660	0.766	0.602	0.518	0.821	0.618	0.195	0.623
GLM-B	0.704 (0.661-0.749)	0.567	0.741	0.464	0.895	0.712	0.749	0.562	0.208	0.631
Internal validation set										
CatBoost	0.766 (0.674-0.857)	0.719	0.754	0.646	0.814	0.660	0.805	0.653	0.195	0.720
LightGBM	0.802 (0.715-0.883)	0.749	0.761	0.771	0.756	0.638	0.855	0.698	0.168	0.538
XGBoost	0.801 (0.717-0.876)	0.743	0.769	0.688	0.814	0.673	0.824	0.680	0.168	0.511
MLP	0.733 (0.635-0.825)	0.659	0.724	0.729	0.721	0.593	0.827	0.654	0.190	0.568
KNN	0.742 (0.647-0.828)	0.676	0.701	0.771	0.663	0.561	0.838	0.649	0.188	0.664
LR	0.726 (0.626-0.818)	0.650	0.739	0.542	0.849	0.667	0.768	0.598	0.202	0.595
AdaBoost	0.805 (0.717-0.879)	0.730	0.724	0.833	0.663	0.580	0.877	0.684	0.207	0.606
GBDT	0.789 (0.701-0.866)	0.710	0.739	0.708	0.756	0.618	0.823	0.660	0.174	0.525
DT	0.760 (0.667-0.843)	0.670	0.776	0.583	0.884	0.737	0.792	0.651	0.193	1.154
ET	0.765 (0.673-0.851)	0.720	0.761	0.521	0.895	0.735	0.770	0.610	0.182	0.546
RF	0.778 (0.687-0.854)	0.708	0.679	0.854	0.581	0.532	0.877	0.656	0.179	0.537
SVM	0.714 (0.612-0.805)	0.652	0.664	0.750	0.616	0.522	0.815	0.615	0.197	0.592
SDT	0.744 (0.659-0.822)	0.607	0.634	0.833	0.523	0.494	0.849	0.620	0.187	0.553
GLM-B	0.662 (0.582-0.737)	0.500	0.724	0.458	0.872	0.667	0.743	0.543	0.225	0.680

Regarding the balance between sensitivity and specificity, RF achieved the highest sensitivity (0.854), but its specificity was relatively low (0.581). ET showed the opposite pattern, with the highest specificity (0.895) but only modest sensitivity (0.521). XGBoost and GBDT provided a more balanced performance in terms of specificity (0.814 and 0.756) and NPV (0.824 and 0.823). AdaBoost achieved a relatively balanced trade-off between sensitivity (0.833) and specificity (0.663), with an overall F1 score of 0.684, a PPV of 0.580, and a high NPV of 0.877, suggesting its potential utility in clinical scenarios where the primary goal is highly sensitive screening of positive cases.

**Figure 5:**
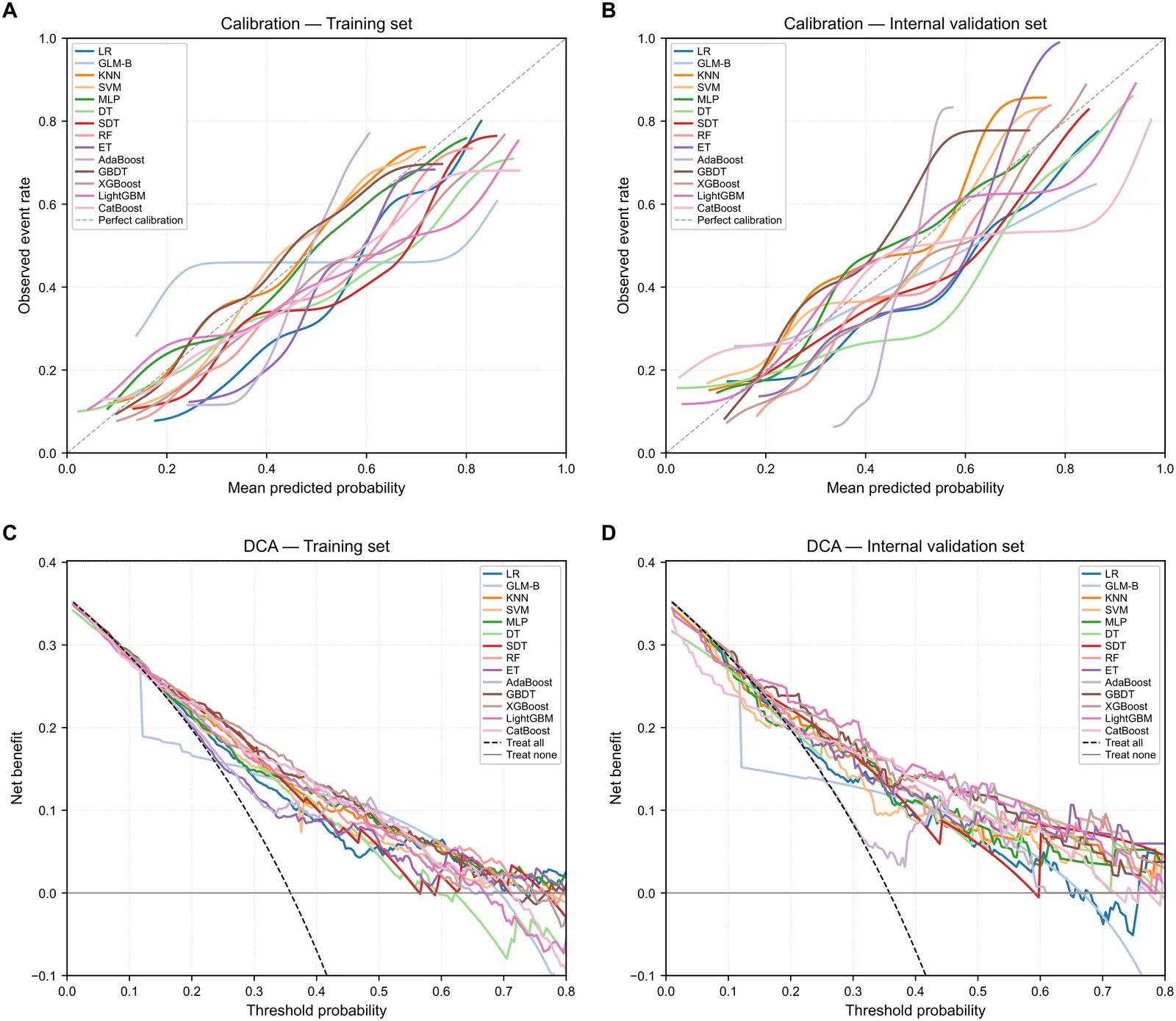
Calibration curves and DCA of the 14 machine learning models in the training set and internal validation set. **(A)** Calibration curves for the training set; **(B)** Calibration curves for the internal validation set; **(C)** DCA for the training set; **(D)** DCA for the internal validation set. The x-axis represents the threshold probability, and the y-axis represents net benefit. The black dashed line indicates the “Treat-all” strategy, and the gray solid line indicates the “Treat-none” strategy.

The calibration curves and DCA of the 14 machine learning algorithms in the training set and internal validation set are shown in **Figure 5**. In terms of calibration (**Figures 5A, B**), boosting-based tree models showed the best overall probability calibration. XGBoost and LightGBM both achieved a Brier score of 0.168 in the internal validation set, followed by GBDT with a Brier score of 0.174. Their calibration curves were closest to the ideal 45° diagonal line, indicating a high degree of agreement between predicted probabilities and observed recurrence rates. RF and CatBoost followed closely, with Brier scores of 0.179 and 0.195. In contrast, although AdaBoost ranked first in AUROC, its Brier score was 0.207, and its calibration curve showed an apparent “probability compression” phenomenon in the middle- and high-probability ranges, deviating notably from the diagonal line. This suggests that the scale of its probability output was systematically shifted, likely attributable to the intrinsic mechanism of the SAMME.R algorithm. LR (Brier = 0.202) and GLM-B (Brier = 0.225) showed mild overestimation in the low-probability range, reflecting the limited ability of linear models to fit nonlinear effects. DT exhibited the most pronounced fluctuation in its calibration curve, consistent with its discretized probability output. In terms of DCA (**Figures 5C, D**), within the clinically acceptable decision-threshold range of 0.10–0.55, the net benefit curves of most ensemble tree models (AdaBoost, XGBoost, LightGBM, GBDT, RF, and CatBoost) were consistently above the two reference lines of “treat-all” and “treat-none,” indicating practical clinical utility. Among them, the net benefit curves of AdaBoost, XGBoost, LightGBM, and GBDT were generally highest within the threshold range of 0.20–0.45, in line with their superior discrimination performance. In contrast, the curves of GLM-B, SVM, and SDT declined rapidly and approached the treat-none line when the threshold exceeded 0.30, suggesting limited net benefit of these models at intermediate-to-high risk thresholds.

Overall, according to the AUROC ranking of the 14 base models in the internal validation set, AdaBoost, LightGBM, and XGBoost ranked in the TOP-3 Together, they constituted a statistically equivalent “top-performing tier”. According to the prespecified optimal model selection strategy, AdaBoost, LightGBM, and XGBoost were identified as the Top-3 base learners and entered the ensemble optimization stage. Their overall performance in terms of AUPRC, calibration, and DCA net benefit further supported this selection.

### 3.5 Ensemble optimization and determination of the optimal model

**Figure 6:**
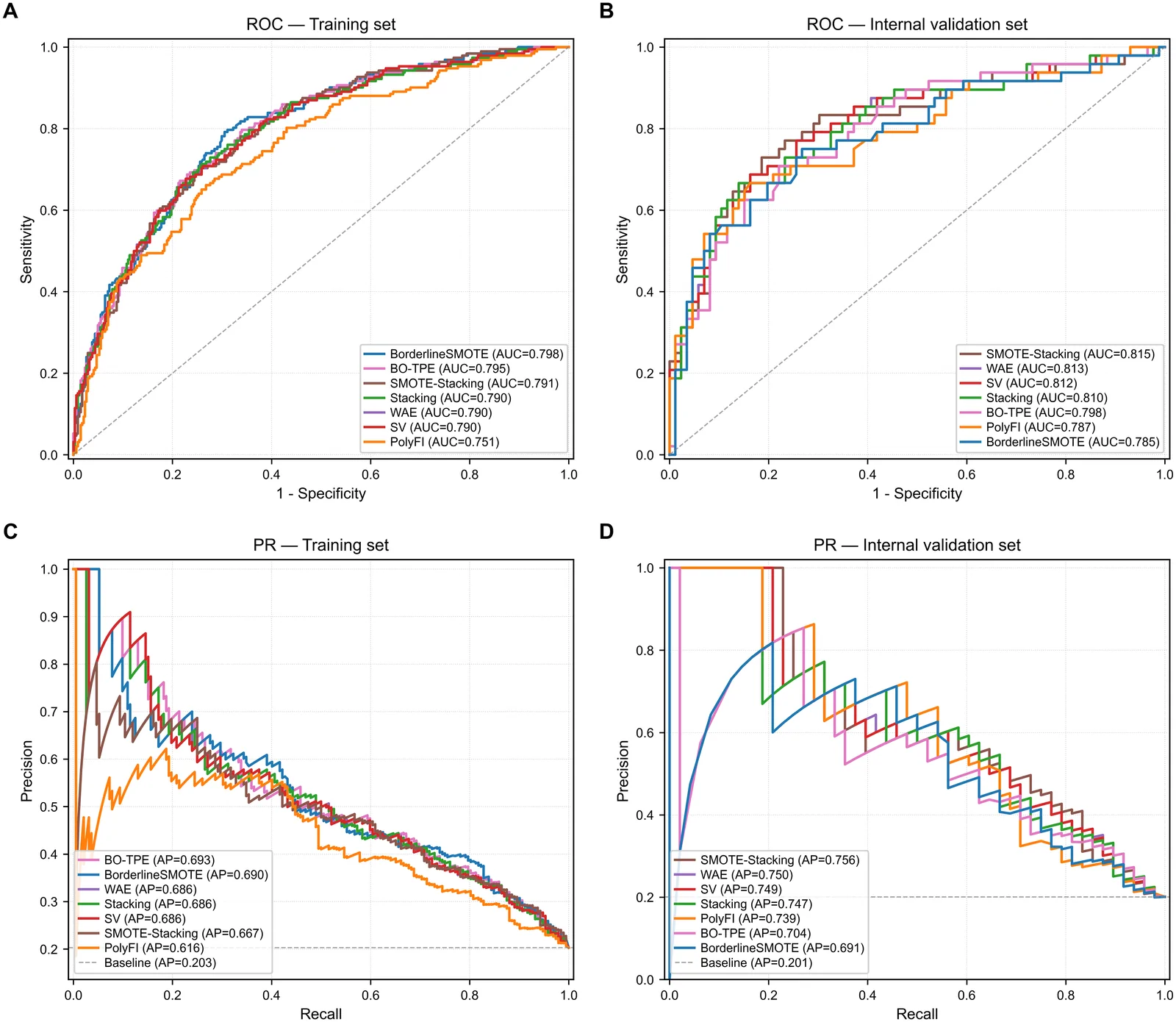
ROC and PR curves of the 7 ensemble optimization strategies on the training set and internal validation set. **(A)** ROC curves for the training set; **(B)** ROC curves for the internal validation set; **(C)** PR curves for the training set; **(D)** PR curves for the internal validation set.

**Figure 7:**
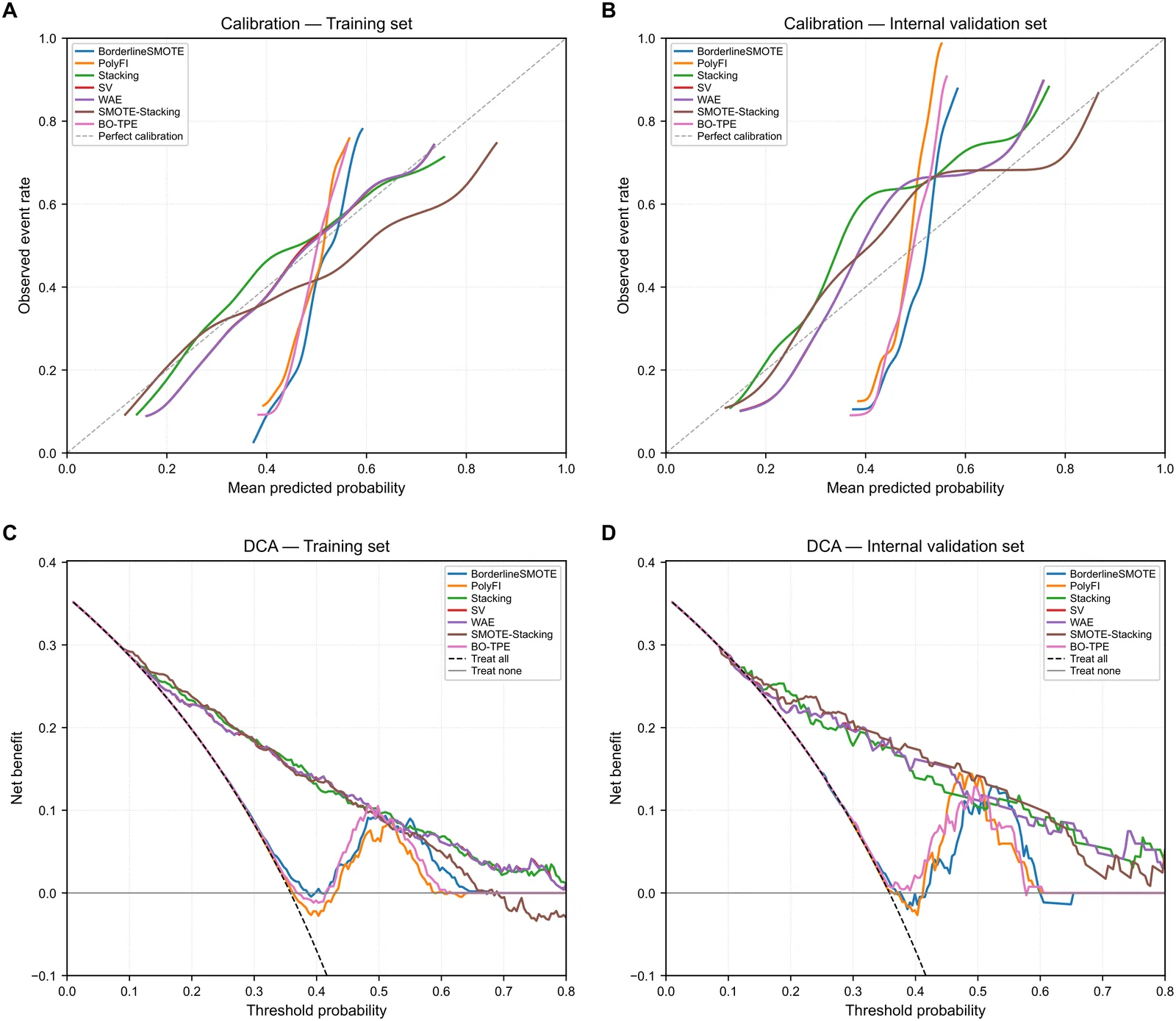
Calibration curves and DCA of the 7 ensemble optimization strategies in the training set and internal validation set. **(A)** Calibration curves for the training set; **(B)** calibration curves for the internal validation set; **(C)** DCA for the training set; **(D)** DCA for the internal validation set.

**Figure 8:**
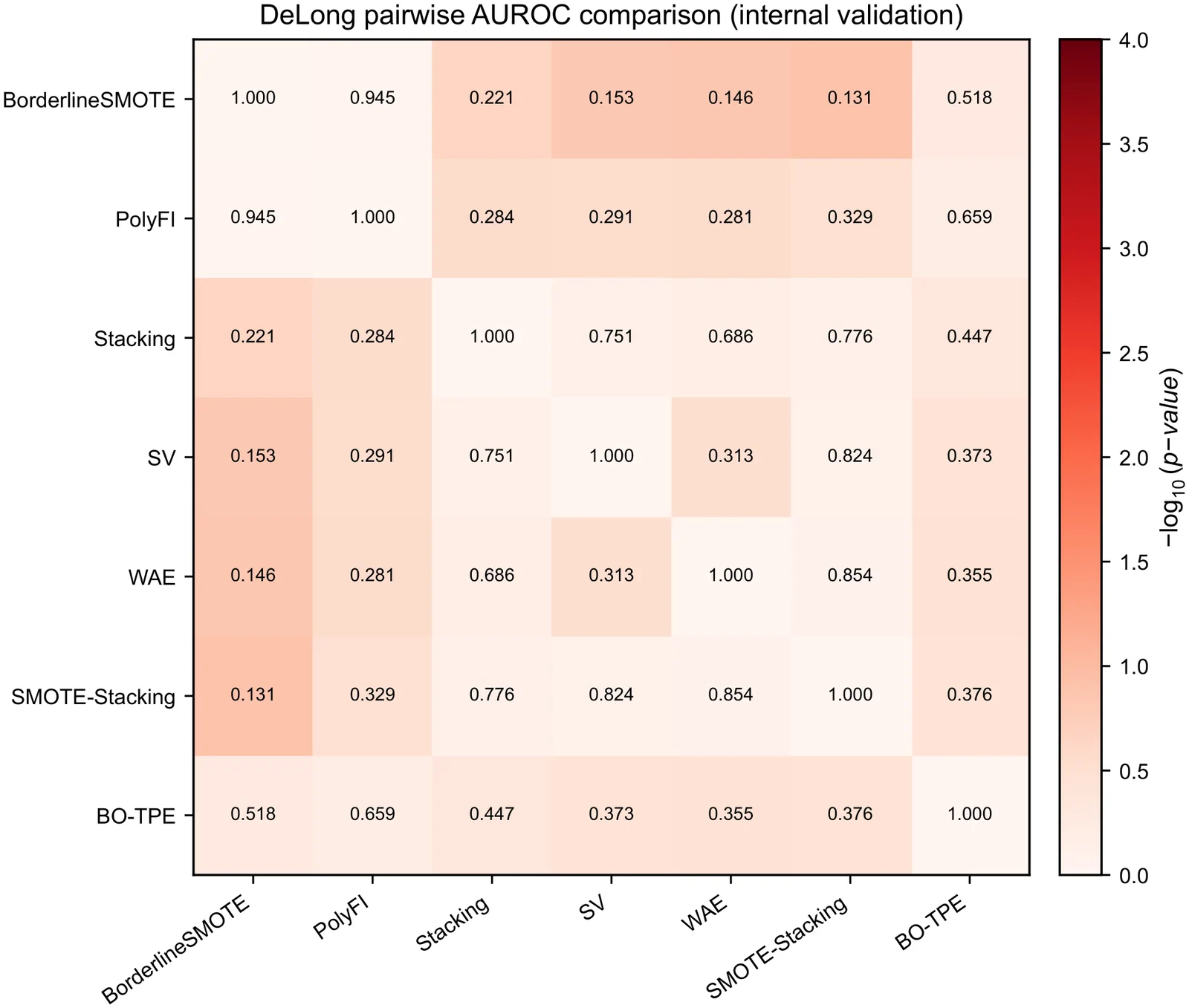
Heatmap of pairwise DeLong comparisons of AUROC among the seven ensemble optimization strategies in the internal validation set.

The performance comparison of the seven ensemble optimization strategies in the training and internal validation sets is presented in **Table 3**, while the ROC curves, PR curves, calibration curves, and DCA are shown in **Figures 6**, **7**, and the heatmap of pairwise DeLong tests is shown in **Figure 8**. In terms of discrimination, SMOTE-Stacking achieved the best performance, with an AUROC of 0.815 (95% CI: 0.726–0.893) and an AUPRC of 0.756 in the internal validation set. WAE (AUROC = 0.813, 95% CI: 0.726–0.889; AUPRC = 0.750), SV (AUROC = 0.812, 95% CI: 0.726–0.888; AUPRC = 0.749), and Stacking (AUROC = 0.810, 95% CI: 0.726–0.888; AUPRC = 0.747) followed closely. The AUROC differences among these four probability-fusion ensemble strategies were all within 0.005, and their 95% CI overlapped substantially. BO-TPE (AUROC = 0.798, 95% CI: 0.711–0.873), PolyFI (AUROC = 0.787, 95% CI: 0.699–0.866), and BorderlineSMOTE (AUROC = 0.785, 95% CI: 0.688–0.869) ranked thereafter. Pairwise DeLong tests showed no statistically significant differences in AUROC among the seven strategies (*p* > 0.05), indicating that they belonged to the same performance range in terms of discrimination. Compared with the original single models, the marginal gain of the ensemble strategies was mainly reflected in probability calibration rather than discrimination. Specifically, the Brier scores of Stacking, SV, WAE, and SMOTE-Stacking were 0.167, 0.166, 0.166, and 0.160, respectively, all lower to varying degrees than those of the Top-3 single models, namely AdaBoost (0.207), LightGBM (0.168), and XGBoost (0.168). Among them, SMOTE-Stacking achieved the lowest Brier score and a log loss of only 0.493, indicating the best agreement between predicted probabilities and observed recurrence probabilities. This finding was consistent with the calibration curves shown in **Figures 7A, B**, in which SMOTE-Stacking and Stacking were closest to the ideal 45° diagonal line, whereas BorderlineSMOTE and BO-TPE showed clear probability overestimation in the low-probability range.

**Table 3:** Performance comparison of the 7 ensemble optimization strategies in the training set and internal validation set.

Model	AUROC (95% CI)	AUPRC	Accuracy	Sensitivity	Specificity	PPV	NPV	F1	Brier	log loss
Training set										
BorderlineSMOTE	0.798 (0.760-0.836)	0.690	0.729	0.781	0.701	0.593	0.852	0.674	0.217	0.626
PolyFI	0.751 (0.708-0.791)	0.616	0.707	0.661	0.733	0.580	0.795	0.618	0.224	0.640
Stacking	0.790 (0.752-0.827)	0.686	0.731	0.714	0.741	0.606	0.823	0.656	0.176	0.528
SV	0.790 (0.752-0.827)	0.686	0.739	0.677	0.773	0.625	0.811	0.650	0.177	0.533
WAE	0.790 (0.752-0.827)	0.686	0.739	0.677	0.773	0.625	0.811	0.650	0.177	0.534
SMOTE-Stacking	0.791 (0.753-0.829)	0.667	0.731	0.703	0.747	0.608	0.818	0.652	0.181	0.539
BO-TPE	0.795 (0.756-0.831)	0.693	0.741	0.688	0.770	0.626	0.815	0.655	0.219	0.630
Internal validation set										
BorderlineSMOTE	0.785 (0.688-0.869)	0.691	0.731	0.667	0.767	0.615	0.805	0.640	0.217	0.627
PolyFI	0.787 (0.699-0.866)	0.739	0.784	0.542	0.919	0.788	0.782	0.642	0.216	0.625
Stacking	0.810 (0.726-0.888)	0.747	0.791	0.604	0.895	0.763	0.802	0.674	0.167	0.510
SV	0.812 (0.726-0.888)	0.749	0.784	0.562	0.907	0.771	0.788	0.651	0.166	0.510
WAE	0.813 (0.726-0.889)	0.750	0.784	0.562	0.907	0.771	0.788	0.651	0.166	0.510
SMOTE-Stacking	0.815 (0.726-0.893)	0.756	0.776	0.583	0.884	0.737	0.792	0.651	0.160	0.493
BO-TPE	0.798 (0.711-0.873)	0.704	0.746	0.625	0.814	0.652	0.795	0.638	0.215	0.622

The ensemble strategies also displayed differing clinical profiles in the sensitivity–specificity trade-off. Under the F1-maximizing threshold (0.49), BorderlineSMOTE achieved the highest sensitivity (0.667) together with relatively balanced performance (F1 = 0.640), making it suitable for recurrence screening scenarios prioritizing a high detection rate. PolyFI (specificity = 0.919, PPV = 0.788), Stacking (specificity = 0.895, PPV = 0.763), and SMOTE-Stacking (specificity = 0.884, PPV = 0.737) demonstrated high specificity and PPV under the Youden threshold, suggesting their suitability for clinical decision-making contexts aimed at avoiding overtreatment. SMOTE-Stacking achieved an overall F1 score of 0.651 and maintained a relatively balanced trade-off between sensitivity (0.583) and specificity (0.884), suggesting good overall usability in recurrence risk stratification.

DCA (**Figures 7C, D**) further showed that within the clinically relevant threshold-probability range of 0.10–0.55, all seven ensemble strategies yielded net benefit curves substantially above the “treat-all” and “treat-none” reference lines. Among them, the net benefit curves of SMOTE-Stacking, WAE, and Stacking remained highest across this interval, indicating robust decision-making utility within clinically acceptable risk thresholds.

Overall, the four leading probability-fusion ensemble strategies (SMOTE-Stacking, WAE, SV, and Stacking) substantially improved probability calibration, whereas their gains in discrimination were relatively limited. Among them, SMOTE-Stacking ranked first in AUROC (0.815), AUPRC (0.756), and Brier score (0.160) by jointly incorporating SMOTE oversampling and a stacking meta-learning mechanism. These models therefore constituted a statistically equivalent “top-performing tier,” and their final suitability for clinical deployment required further confirmation based on cross-cohort robustness in the external validation cohort.

### 3.6 External validation results

**Figure 9:**
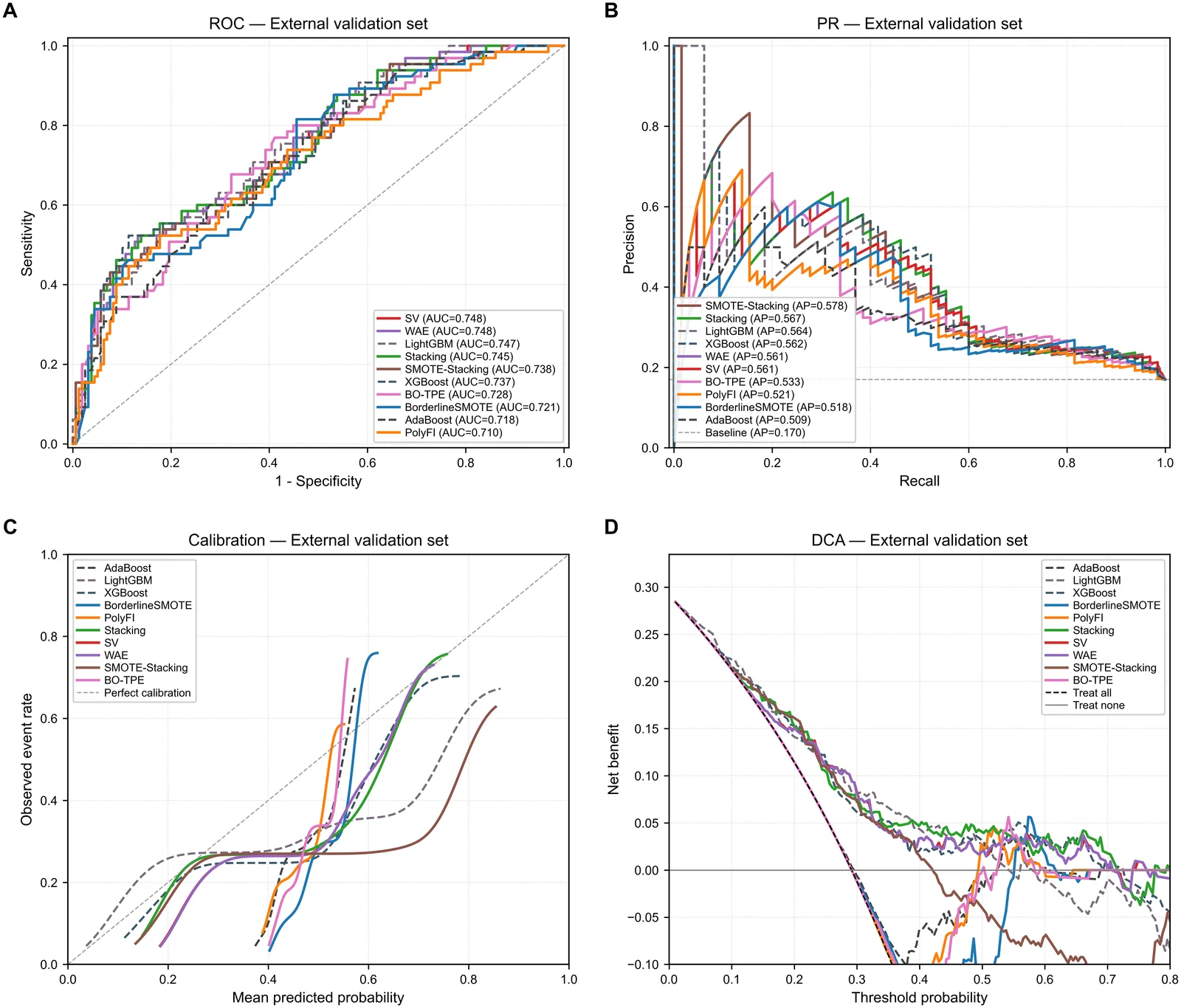
Comprehensive performance evaluation of the Top-3 single base learners and the seven ensemble optimization strategies in the external validation cohort. **(A)** ROC curves; **(B)** PR curves; **(C)** calibration curves; **(D)** DCA.

To evaluate cross-center generalizability, the seven ensemble optimization models and the Top-3 single base learners were directly applied to the external validation cohort. The external validation performance of each model is summarized in **Table 4**. In terms of discrimination (**Figures 9A, B**), SV and WAE performed jointly best, both achieving an AUROC of 0.748 (95% CI: 0.682–0.813 and 0.681–0.813). Among the single base learners, LightGBM achieved an external AUROC of 0.747 (95% CI: 0.681–0.815), only 0.001 lower than SV/WAE and slightly outperforming Stacking (0.745). Stacking, SMOTE-Stacking, XGBoost, and BO-TPE followed closely, with AUROCs ranging from 0.728 to 0.745, whereas BorderlineSMOTE, AdaBoost, and PolyFI ranked lowest. In terms of AUPRC, SMOTE-Stacking ranked first (0.578), followed by Stacking (0.567), LightGBM (0.564), XGBoost (0.562), and SV/WAE (0.561). Pairwise DeLong tests across all 45 model pairs showed no statistically significant differences in external AUROC (*p*> 0.05), indicating that the Top-3 single models and the seven ensemble strategies fell within the same performance range in terms of discrimination on the independent cohort.

**Table 4:** Performance comparison of the TOP-3 single base learners and the seven ensemble optimization strategies in the external validation cohort.

Model	AUROC (95% CI)	AUPRC	Accuracy	Sensitivity	Specificity	PPV	NPV	F1	Brier	Log loss	Threshold
AdaBoost	0.718 (0.644-0.785)	0.509	0.596	0.723	0.544	0.395	0.827	0.511	0.219	0.630	0.455
LightGBM	0.747 (0.681-0.815)	0.564	0.717	0.569	0.778	0.514	0.815	0.540	0.188	0.554	0.433
XGBoost	0.737 (0.668-0.804)	0.562	0.695	0.554	0.753	0.480	0.804	0.514	0.183	0.544	0.447
BorderlineSMOTE	0.721 (0.648-0.789)	0.518	0.596	0.723	0.544	0.395	0.827	0.511	0.233	0.659	0.490
PolyFI	0.710 (0.637-0.782)	0.521	0.668	0.538	0.722	0.443	0.792	0.486	0.223	0.638	0.484
Stacking	0.745 (0.677-0.811)	0.567	0.637	0.615	0.646	0.417	0.803	0.497	0.179	0.537	0.339
SV	0.748 (0.682-0.813)	0.561	0.682	0.615	0.709	0.465	0.818	0.530	0.183	0.547	0.412
WAE	0.748 (0.681-0.813)	0.561	0.682	0.615	0.709	0.465	0.818	0.530	0.183	0.547	0.413
SMOTE-Stacking	0.738 (0.666-0.806)	0.578	0.632	0.646	0.627	0.416	0.811	0.506	0.212	0.610	0.422
BO-TPE	0.728 (0.652-0.796)	0.533	0.664	0.600	0.690	0.443	0.807	0.510	0.224	0.641	0.484

With respect to calibration (**Figure 9C**), Stacking and XGBoost demonstrated the best agreement between predicted and observed probabilities, with external Brier scores of 0.179 and 0.183 and log loss of 0.537 and 0.544. Their calibration curves were closest to the ideal diagonal line over the probability range of 0.20–0.55. SV, WAE, and LightGBM followed closely, with Brier scores ranging from 0.183 to 0.188. Notably, the Brier score of SMOTE-Stacking increased from 0.160 in internal validation to 0.212 externally, indicating substantial calibration deterioration, which may reflect covariate shift between SMOTE-generated synthetic samples and the true external distribution. AdaBoost and its derived strategies, BorderlineSMOTE and BO-TPE, showed further probability overestimation in the external cohort (Brier 0.219–0.233), consistent with amplification of the probability compression characteristic of the AdaBoost SAMME.R algorithm under cross-center conditions.

In the DCA (**Figure 9D**), within the clinically relevant threshold range of 0.10–0.45, the net benefit curves of SV, WAE, Stacking, and LightGBM remained highest and overlapped substantially, lying clearly above the “treat-all” and “treat-none” reference lines across the 0.20–0.40 interval. XGBoost and SMOTE-Stacking provided comparable net benefit within this interval. By contrast, AdaBoost, BorderlineSMOTE, and BO-TPE showed rapidly declining net benefit beyond a threshold of 0.40, ultimately falling below zero.

In terms of cross-cohort generalization stability, compared with internal validation (AUROC 0.785–0.815), external AUROC values generally declined by 0.05–0.10. LightGBM showed the most stable cross-cohort performance, followed by XGBoost. The attenuation observed in SV, WAE, Stacking, and SMOTE-Stacking ranged from 0.064 to 0.077. AdaBoost exhibited the greatest decline, and its derived strategies BorderlineSMOTE and BO-TPE likewise showed marked cross-cohort deterioration. These findings suggest that when external data exhibit covariate shift, ensemble fusion strategies do not necessarily confer additional gains in generalizability relative to a single base learner; rather, their advantages may lie more in probability calibration and net benefit.

Considering discrimination, calibration, decision-curve net benefit, and cross-cohort generalization stability together, SV demonstrated the most robust overall performance in the external validation cohort. In contrast, although SMOTE-Stacking achieved the highest discrimination in internal validation, it showed evident calibration deterioration and discriminatory decline, suggesting that the probability boundaries learned from SMOTE-generated synthetic samples may have limited generalizability across centers. According to the second-stage confirmation principle prespecified in the optimal model selection strategy, SV was identified as the final candidate model for clinical deployment in this study and was subsequently selected as the target model for SHAP interpretability analysis.

### 3.7 SHAP interpretability analysis

**Figure 10:**
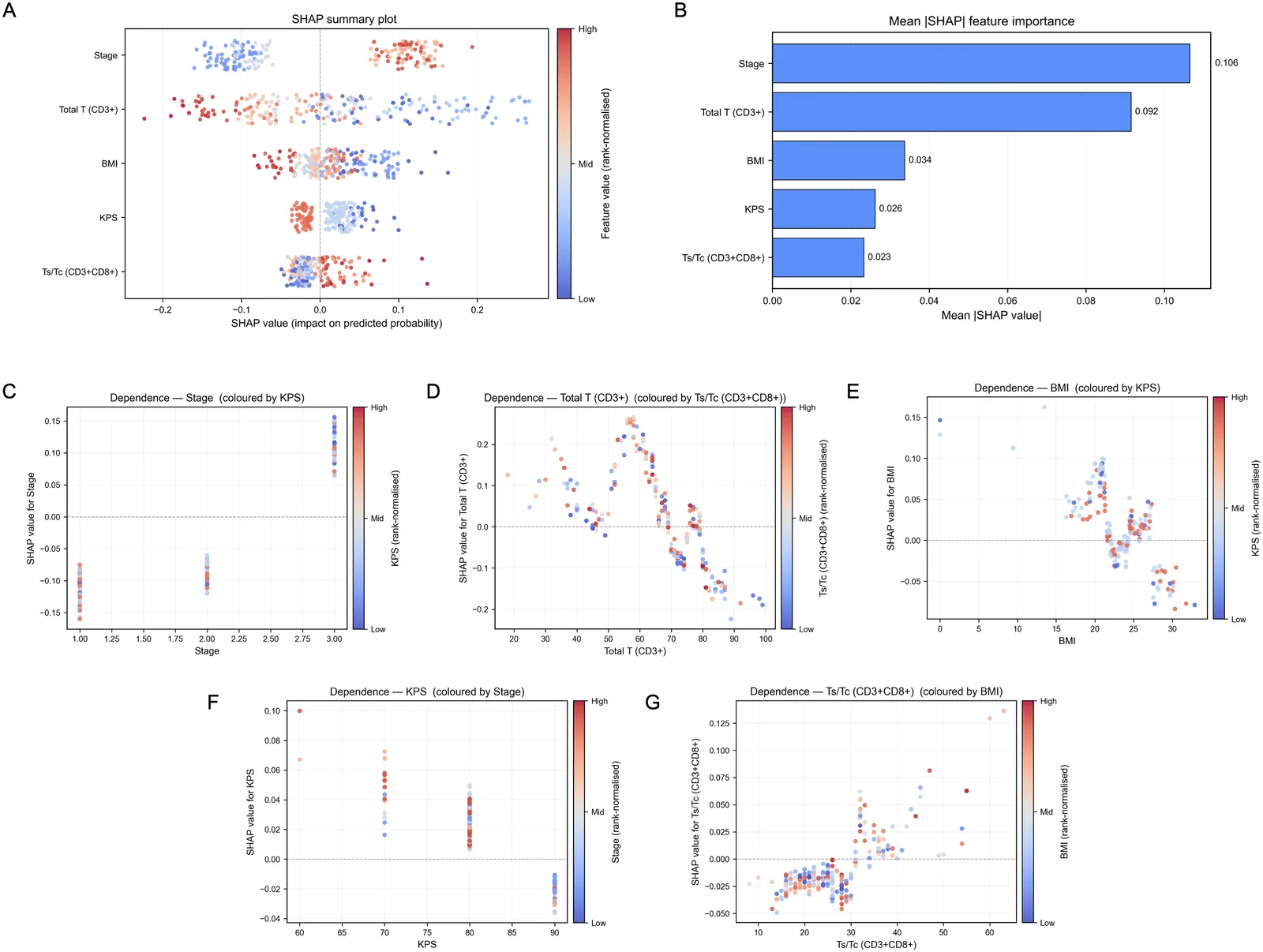
SHAP interpretability analysis of the SV model. **(A)** SHAP beeswarm plot for the external validation cohort. Each dot represents one patient. The x-axis shows each feature’s SHAP contribution to the predicted probability of recurrence, and the color encodes the feature value from low (blue) to high (red) after rank normalization. Features are ordered by mean |SHAP|, with the most important at the top; **(B)** bar plot of mean |SHAP| feature importance, quantifying the overall contribution of each predictor at the population level; **(C–G)** dependence plots arranged in descending order of feature importance. The x-axis represents the feature value, and the y-axis represents the corresponding SHAP value. Dot color encodes the value of the most strongly interacting feature, highlighting interaction effects.

In this study, SHAP was used to interpret the SV model, and the results were reported from four perspectives: global feature contribution, nonlinear relationships between features and model predictions, individual decision pathways, and population-level risk stratification. Global feature importance analysis showed (**Figures 10A, B**) that among the five predictors retained after the dual feature selection strategy combining Boruta and LASSO, stage was the single most important variable (mean |SHAP| = 0.106), followed closely by Total T (0.092). The remaining three variables, namely BMI (0.034), KPS (0.026), and Ts/Tc (0.023), made substantially smaller contributions, although they should not be disregarded. Notably, lymphocyte subset-related indicators accounted for approximately 41% of the cumulative mean |SHAP| signal, highlighting the explanatory value carried by peripheral blood immunological features in predicting postoperative lung cancer recurrence. The beeswarm plot revealed coherent and biologically plausible directional effects across all predictors: more advanced stage and lower Total T shifted SHAP values toward the positive side, thereby increasing the predicted probability of recurrence. Similarly, lower BMI, lower KPS, and higher Ts/Tc were also associated with elevated recurrence risk. Dependence plots (**Figures 10C–G**) further characterized these patterns in greater detail. Stage exhibited a marked stepwise threshold effect, with SHAP values for stage III cases consistently clustering around +0.10, whereas stages I–II showed negative contributions of comparable magnitude (**Figure 10C**). Total T demonstrated an approximately linear inverse relationship with its SHAP contribution: patients with CD3+ percentages below about 50% received substantially positive risk attribution, whereas values above about 70% systematically decreased the predicted recurrence probability (**Figure 10D**). The remaining three predictors, namely BMI, KPS, and Ts/Tc, also followed clinically expected gradients without anomalous inflection points.

**Figure 11:**
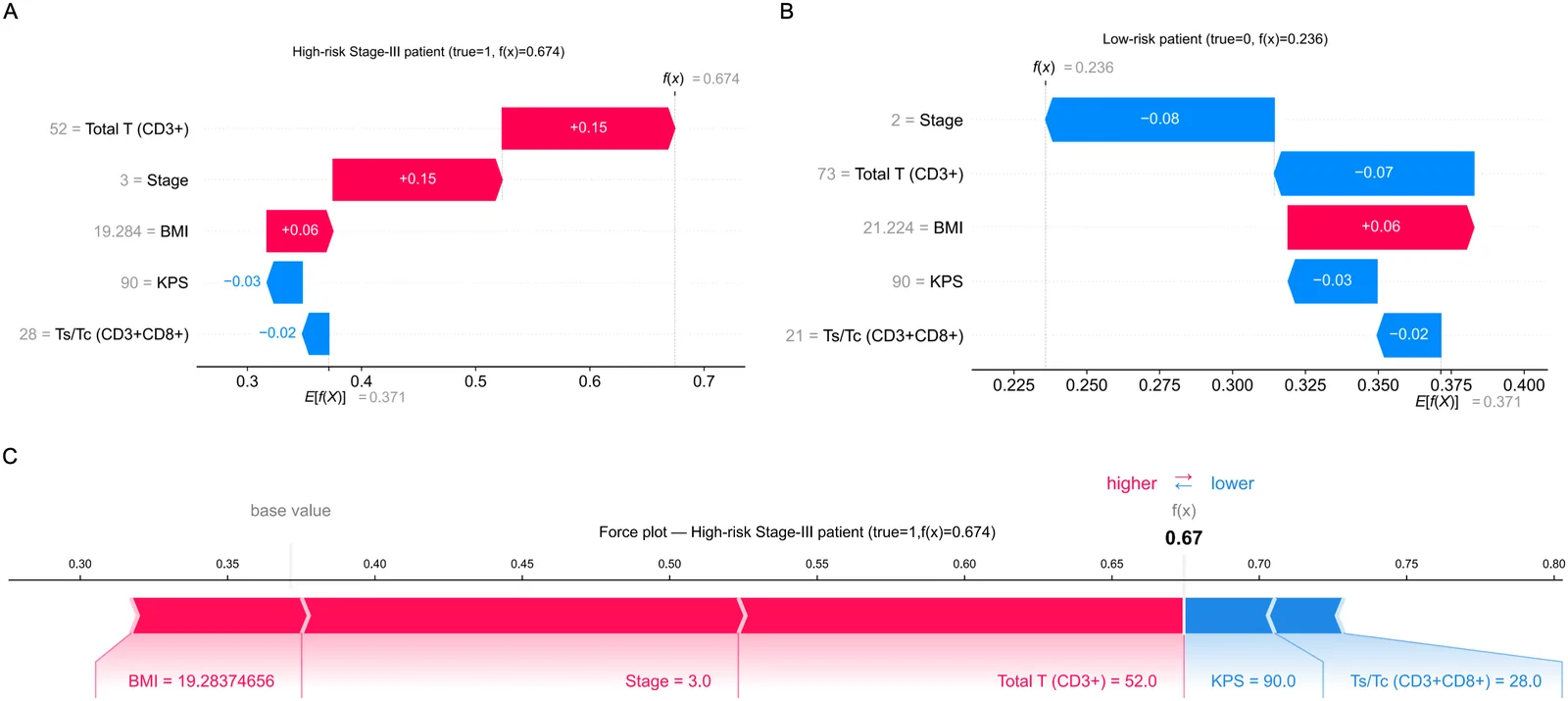
Representative Case Analysis. **(A)** Waterfall plot for a high-risk true-positive patient (true = 1, f(x) = 0.674); **(B)** waterfall plot for a low-risk true-negative patient (true = 0, f(x) = 0.236); **(C)** SHAP force plot for the high-risk true-positive patient. Red arrows indicate feature contributions that increase the predicted probability, whereas blue arrows indicate feature contributions that decrease the predicted probability.

At the individual level, two representative cases were selected from the external validation cohort for individualized decomposition. One stage III patient (BMI = 19.28 kg/m², KPS = 90, Total T = 52%, Ts/Tc = 28%; **Figures 11A, C**) was correctly classified as high risk (f(x) = 0.674; true outcome: recurrence). The waterfall plot showed that low Total T (+0.15) and advanced stage (+0.15) jointly dominated risk elevation, while low BMI (+0.06) further increased the predicted probability, only partially offset by favorable KPS (−0.03) and median Ts/Tc (−0.02). The force plot presented the same process as a sequence of cumulative forces: the predicted probability was continuously pushed upward from the population baseline E[f(X)] = 0.371 to f(x) = 0.674 by the combined effects of immune status, stage, and nutritional status. In contrast, a low-risk reference case (stage II, BMI = 21.22, KPS = 90, Total T = 73%, Ts/Tc = 21%; **Figure 11B**) showed concordant negative contributions from stage (−0.08), Total T (−0.07), KPS (−0.03), and Ts/Tc (−0.02), with only BMI (+0.06) exerting a mild reverse effect, resulting in a final predicted probability of 0.236, consistent with the patient’s true non-recurrence outcome.

Taken together, the SHAP interpretability analysis indicated that recurrence risk prediction by the SV model in elderly NSCLC was driven primarily by the two major clinical variables, Stage and Total T, with BMI, KPS, and Ts/Tc serving as auxiliary modifying factors. The direction of model decision-making was consistent with established clinical and immunological knowledge, and the feature contribution patterns were highly reproducible across both the internal and external cohorts, further supporting the clinical credibility and translational value of the SV model as the final predictive model in this study.

## 4 Discussion

Global trend analyses spanning 1990 to 2021 have demonstrated a significant rise in lung cancer incidence among the elderly population (51). Although surgery is regarded as the most effective treatment for patients with early-stage NSCLC, including elderly patients (52), postoperative recurrence remains the leading determinant of poor long-term survival in this group. The temporal pattern of recurrence is characterized by multiple peaks, and recurrence risk varies dynamically across postoperative time intervals, with 6–9 months and 18–24 months after surgery representing high-risk periods for recurrence (53, 54). One study found that in patients aged 75 years or older who underwent complete resection for NSCLC, the 2-year recurrence rate was about 29.2% (55). Accordingly, precise prediction of recurrence within 2 years after surgery is particularly important. In elderly patients with lung cancer, immune cell alterations in the setting of immunosenescence may provide a more sensitive signal of recurrence propensity driven by postoperative minimal residual lesions. To date, multiple studies have evaluated the prognostic significance of tumor-infiltrating lymphocytes (TIL) in lung cancer (56, 57), yet assessment of the local immune microenvironment is limited by several clinical disadvantages, including invasiveness, labor intensity, and the impracticality of repeated measurements. As key indicators of systemic immune surveillance and antitumor immunity, peripheral blood T-lymphocyte subsets have increasingly attracted attention in cancer prognostic assessment because they are readily accessible and amenable to longitudinal monitoring (58).

This study successfully developed a prediction model by identifying risk factors for ER. The results showed that 136 patients (20.30%) in the development cohort and 38 patients (17.0%) in the external validation cohort developed ER within 2 years after surgery, further confirming that elderly patients indeed face a substantial risk of recurrence and metastasis. Based on the clinicopathological characteristics and peripheral blood T-lymphocyte subset indicators of elderly postoperative lung cancer patients, and guided by current guidelines, consensus statements, and expert clinical experience, 23 candidate predictors were initially proposed. These variables were subsequently reduced to 5 final predictors, namely tumor stage, total T-lymphocyte percentage, BMI, KPS, and Ts/Tc percentage, using a dual feature selection strategy combining LASSO regression and the Boruta algorithm. To construct the clinical prediction model, we first systematically evaluated 14 machine learning algorithms and 7 ensemble optimization strategies for ER prediction. In the base-model stage, AdaBoost, LightGBM, and XGBoost were selected as the TOP-3 base learners for the ensemble optimization stage. Among the 7 ensemble optimization strategies, the probability-fusion models (SMOTE-Stacking, WAE, SV, and Stacking), ranked in the top four in terms of AUROC in the internal validation set (0.815, 0.813, 0.812, and 0.810), and their corresponding Brier scores (0.160–0.167) were markedly lower than those of the single models, indicating that the main benefit of the fusion strategies lay in improving probability calibration. When the 7 ensemble optimization models and the TOP-3 single base learners were directly applied to the external validation cohort (n = 223), the external AUROC of SV was 0.748 (95% CI: 0.682–0.813), showing only slight attenuation compared with internal validation (ΔAUC = 0.064). Its performance fell within the same range as that of WAE (0.748), LightGBM (0.747), and Stacking (0.745), and paired DeLong tests showed no statistically significant pairwise differences in external AUROC. Considering discrimination, calibration, decision-curve net benefit, and cross-cohort generalization stability together, SV demonstrated the most robust overall performance in the external validation cohort. This attenuation may be attributable to the following factors: First, marked baseline differences were present between the two centers: compared with the development cohort, the external cohort had a higher proportion of male patients (69.1% vs 51.7%), a lower proportion of adenocarcinoma (54.7% vs 72.0%), a lower median KPS score (80 vs 90), and a different comorbidity profile, including lower rates of hypertension (23.7% vs 44.2%), coronary heart disease (4.9% vs 17.9%), and family history of cancer (6.8% vs 25.9%). These differences may have altered the marginal effects and weighting of some predictors. Second, Although T-lymphocyte subset measurements at each clinical laboratory were performed according to standardized operating procedures, including routine instrument calibration, internal quality control, and the same flow cytometric gating criteria, the platforms, reagent lots, and instrument settings used across the participating centers were not prospectively harmonized. Systematic differences in T-lymphocyte subset measurements across testing platforms may have been magnified under cross-center application, as exemplified by the lower median Ts/Tc value in the external cohort than in the training set (25.94 vs 28.47, *p* < 0.001). Of note, compared with the single AdaBoost model (external AUROC = 0.718, ΔAUC = 0.087), the Top-3 probability-fusion strategies: SV, WAE, Stacking, and SMOTE-Stacking, all with external ΔAUC values between 0.064 and 0.077, showed more stable cross-center generalization. Furthermore, SHAP analysis clarified both the direction and magnitude of the contributions of each predictor within the SV model. Tumor stage and Total T emerged as the two major drivers of prediction, with mean |SHAP| values of 0.106 and 0.092. Meanwhile, lymphocyte subset-related indicators (Total T and Ts/Tc) together accounted for approximately 41% of the cumulative mean |SHAP| signal, suggesting that integrated assessment of peripheral blood T-lymphocyte subsets and clinicopathological features may offer greater clinical value for predicting ER risk.

The discrepancy between the internal and external calibration performance of SMOTE-Stacking also warrants consideration. Although SMOTE-Stacking achieved the best performance in internal validation, its Brier score increased from 0.160 in internal validation to 0.212 in external validation. SMOTE generates synthetic minority-class observations by interpolating between neighboring samples in the training feature space, which helps alleviate class imbalance and facilitates the identification of minority-class patterns during model development. However, these synthetic observations may not fully reproduce the joint covariate distributions observed in independent clinical populations and may amplify center-specific feature relationships, resulting in a mismatch between the distribution learned during training and that of the external population. This mechanism may explain why SMOTE-Stacking retained good discriminative ability during cross-center transfer but showed poorer calibration, consistent with the observed deterioration in its external Brier score. In contrast, SV approach averages the predicted probabilities of the Top-3 base learners without generating synthetic patients, which may partly explain its more stable calibration performance in the external cohort.

Among the predictive factors, tumor stage remains the primary determinant of ER. A higher stage usually indicates a broader extent of primary tumor invasion, more severe lymph node involvement, and a greater burden of potential micrometastases. Consequently, even after surgical resection, patients are still more likely to retain microscopic residual lesions that cannot be detected by conventional methods, thereby increasing the risk of ER (59). Kawachi et al. analyzed 402 patients with stage I NSCLC who underwent surgery and found that 10.7% developed ER (60). Zhao et al. retrospectively analyzed 186 patients with stage IA–IIA NSCLC treated surgically, of whom 15.6% experienced ER (61). Kiankhooy et al. followed 532 patients with lymph node-negative T1–T2b lung cancer and reported an ER rate of 19% (62). According to data from Wenzhou Medical University, when tumor stage reached IIIA, 28.99% of patients with NSCLC experienced recurrence within 1 year after curative resection (63). With increasing stage, the probability of recurrence within 1 year may rise to 38.3%–47% (64, 65). Regrettably, our study showed that T stage and N stage were not statistically significant in predicting ER, possibly because they are closely correlated with stronger predictive factors such as overall tumor stage. When these more robust predictors are included in a multivariable model, the independent effects of T stage and N stage may be attenuated.

In addition, BMI and KPS were also of considerable importance in recurrence risk. Our results showed that low BMI was a risk factor for ER, which is consistent with the findings of Fukumoto et al. from a retrospective analysis of 16,503 cases in a database (66). Khan et al. further suggested that postoperative loss of skeletal muscle volume is an important contributor to low BMI and ultimately promotes recurrence after resection of early-stage NSCLC (67). KPS is a classic indicator used to assess physical function and performance status in patients with lung cancer, and substantial evidence has shown that KPS is an independent prognostic factor for postoperative recurrence and overall survival (68, 69). Patients with lower KPS scores are usually accompanied by more severe systemic inflammatory consumption and immunosuppression, which may impair the postoperative control of micrometastatic lesions in peripheral blood or lymph nodes and thereby increase the risk of ER.

The most distinctive finding of this study is that several T-lymphocyte-related indicators entered the final model, including the percentage of total T and Ts/Tc. This suggests that ER is not driven by a single immune abnormality but is more likely to reflect a multilevel imbalance in T-lymphocyte immunity. This imbalance may be particularly pronounced in elderly patients in the context of immunosenescence. The total T-lymphocyte percentage can reflect the overall cellular immune reserve of the host (70, 71). Abnormal total T-lymphocyte levels often indicate impaired immune surveillance, reducing the host’s ability to recognize and eliminate postoperative residual tumor cells and micrometastatic lesions, thereby increasing the likelihood of recurrence (72). Elderly patients are intrinsically affected by immunosenescence, characterized by thymic involution, delayed immune responses, and disrupted immune homeostasis. Therefore, changes in the proportion of total T-lymphocytes may have stronger risk-indicating significance in this population.

According to the structure of the T-cell receptor, T cells can be classified into αβ and γδ subsets, with the αβ subset further divided into CD4+ T cells and CD8+ T cells (73). Notably, the percentage of CD8+ T cells was also selected as a predictor, indicating that functional imbalance among T-lymphocyte subsets may represent an important immunological basis for ER. Although CD8+ T cells are the principal effector cells responsible for directly killing tumor cells, their prognostic significance in lung cancer remains controversial. Some studies have shown that CD8+ T cells are associated with favorable prognosis in NSCLC (74). However, other studies have reported CD8+ T cells as an adverse prognostic factor (75, 76). In our study, a higher CD8+ T-cell level appeared to promote ER in elderly patients with lung cancer. We speculate that there may be two explanations. First, CD8+ T cells are highly heterogeneous (77). Based on the surface marker CD28, CD8+ T cells can be further subdivided into suppressor T cells and cytotoxic T cells, CD8+CD28− is a surface phenotype of suppressor T cells and is associated with impaired immune function (78). Second, under persistent stimulation by tumor antigens, CD8+ T cells may gradually lose effector function, show reduced proliferative capacity, and highly express multiple inhibitory receptors. These changes substantially weaken the antitumor effects mediated by granzyme and perforin release from CD8+ T cells (79, 80). Therefore, although peripheral blood CD8+ T-cell levels were higher in the ER group, this population may have contained more functionally suppressed or nonfunctional cells, with fewer CD8+ T cells truly exerting antitumor activity. Regrettably, CD4+ T-cells and Treg were not identified as significant independent predictors of ER in elderly patients with lung cancer, which may be related to the limited sample size of this study.

Overall, the SV model developed in this study showed favorable discrimination (AUROC = 0.812) and calibration (Brier score = 0.166) in internal validation, and achieved an AUROC of 0.748 in external validation, with relatively robust cross-center generalizability (ΔAUC = 0.064). These findings indicate that a probability fusion strategy built upon the Top-3 boosting-based base learners may help mitigate the impact of cross-center covariate shift. This result further indicates that future model implementation may benefit from center-specific recalibration, threshold optimization, or multimodel probability fusion strategies to improve inter-institutional applicability. From the perspective of model development, our findings suggest that relying solely on conventional clinicopathological variables may be insufficient for ER risk assessment in elderly patients with lung cancer. Compared with imaging and pathological indicators, peripheral blood T-lymphocyte subset profiling offers several advantages, including noninvasiveness, accessibility, repeatability, and convenience for dynamic monitoring. The incorporation of T-lymphocyte subsets therefore enables a more comprehensive characterization of patient risk profiles and provides new insight for individualized follow-up and intervention in elderly patients with lung cancer.

This study also has several limitations. First, our classification of T-lymphocytes was relatively coarse and did not capture more functionally defined T-lymphocyte subsets, including naive and memory T cells, thereby restricting our ability to understand the functional dynamics of the immune response. Future work could apply high-resolution approaches, such as single-cell RNA sequencing, to delineate the functional heterogeneity of T-lymphocytes. Second, the immune variables incorporated in this study were predominantly percentage-based indicators, without further integration of absolute cell counts or longitudinal changes. Relative percentages may be influenced by variations in total leukocyte or lymphocyte counts, particularly in older populations, thereby limiting the interpretation of the underlying immune mechanisms. In addition, ER was treated as a fixed binary outcome. Although no right censoring occurred within the 2-year window, binary classification does not account for the exact timing of recurrence and may therefore obscure differences between very early and later recurrence within this interval. Future studies should consider time-to-event machine-learning approaches, such as Cox-LASSO or Random Survival Forests, to more precisely characterize recurrence risk over time. Finally, although external validation was performed, the stability and generalizability of the model should be further evaluated in larger, multicenter populations.

## 5 Conclusion

This study demonstrates that tumor stage, total T-lymphocyte percentage, BMI, KPS, and Ts/Tc percentage are important predictors of ER in elderly patients with lung cancer. Collectively, these factors suggest that ER in this population reflects the joint contribution of tumor burden, host performance status, and immune dysregulation. The prediction model constructed using these variables may serve as an effective tool for postoperative risk stratification and individualized management, while also providing a basis for further exploration of the immunological mechanisms of ER in elderly patients with lung cancer.

## Data Availability

The original contributions presented in the study are included in the article/**Supplementary Material**. Further inquiries can be directed to the corresponding author.

## Glossary

AdaBoost: Adaptive boosting
AUPRC: Area under the precision-recall curve
AUROC: Area under the receiver operating characteristic curve
BMI: Body mass index
BO-TPE: Bayesian Hyperparameter Optimization
BSA: Body surface area
CatBoost: Categorical boosting
CHD: Coronary heart disease
COPD: Chronic obstructive pulmonary disease
CT: Computed tomography
DCA: Decision Curve Analysis
DT: Decision tree
EPV: Events Per Variable
ER: Early recurrence
ET: Extremely randomized tree
FCM: Flow cytometry
GBDT: Gradient boosting decision tree
GLM-B: Generalized linear model with binomial distribution
KNN: K-nearest neighbors
KPS: Karnofsky performance scale
LASSO: Least absolute shrinkage and selection operator
LightGBM: Light gradient boosting machine
LR: Logistic regression
MICE: Multiple imputation by chained equations
MLP: Multilayer perceptron
MRI: Magnetic resonance imaging
NPV: Negative predictive value
NSCLC: Non-small cell lung cancer
OOF: Out-of-fold
PolyFI: Polynomial Feature Interaction
PPV: Positive predictive value
RF: Random forest
SDT: Shallow decision tree
SHAP: Shapley additive explanations
SMOTE-Stacking: SMOTE-augmented Stacking Ensemble
SV: Soft Voting Ensemble
SVM: Support vector machine
Th: Helper/inducer T-lymphocyte
TIL: Tumor-infiltrating lymphocytes
Treg: Regulatory T cells
Ts/Tc: Suppressor/cytotoxic T-lymphocyte
VATS: Video-assisted thoracic surgery
WAE: Weighted Averaging Ensemble
XGBoost: Extreme gradient boosting.
