# Supplementary material for: Peripheral blood T-lymphocyte subsets provide prediction of early postoperative recurrence in elderly patients with lung cancer: model development and external validation
Source: Front Oncol. 2026 Sep 7;16:1948080. doi: 10.3389/fonc.2026.1948080 (PMC13591314; doi:10.3389/fonc.2026.1948080)
Supplement: Supplementary file 1 [file DataSheet1.docx]

**Flow cytometry (FCM) analysis of Peripheral Blood T-Cell Subsets**

1. Preparation of solutions: Prepare 1× red blood cell lysis buffer, mix thoroughly by vortexing, and keep at room temperature until use.

2. Under light-protected conditions, add the corresponding fluorochrome-conjugated antibodies against cell-surface markers or the respective isotype control antibodies to each tube. The antibody volumes used in each tube are shown in Table 1.

**Table 1. Antibody or isotype-control volumes for CD45, CD3, CD4, CD8, CD25, and CD127**

| Surface marker | CD45 | CD3 | CD4 | CD8 | CD25 | CD127 |
| --- | --- | --- | --- | --- | --- | --- |
| Fluorescence channel | V500-C | APC-H7 | PE-Cy7 | PerCP | APC | PE |
| 1 | Antibody,  5 μL | Antibody,  5 μL | Antibody,  5 μL | Antibody,  20 μL | Isotype control,  5 μL | Isotype control,  20 μL |
| 2 | Antibody,  5 μL | Antibody,  5 μL | Antibody,  5 μL | Antibody,  20 μL | Antibody,  5 μL | Antibody,  20 μL |

3. After thorough mixing, 100 μL of EDTA-anticoagulated peripheral blood was added to each tube containing the corresponding antibodies. The samples were vortexed and incubated for 15 min at room temperature in the dark.

4. After incubation, 2 mL of 1× red blood cell lysis buffer was added to each tube. The samples were vortexed and incubated at room temperature in the dark until complete erythrocyte lysis was achieved, as indicated by complete clearing of the sample, which generally required approximately 10 min.

5. The samples were centrifuged at 1,300 rpm for 5 min, and the supernatant was discarded. Subsequently, 2 mL of PBS was added to each tube for washing, followed by centrifugation at 1,300 rpm for 5 min. The supernatant was then discarded.

6. The cell pellet in each tube was resuspended in 300 μL of PBS.

7. T-cell subsets were analyzed using a flow cytometer.

**FCM Gating Strategy (Figure 1)**

1. Single cells were first identified using forward scatter height (FSC-H) versus forward scatter area (FSC-A), and the P1 gate was established to select events with corresponding area and height characteristics. Cellular debris and dead cells were excluded to minimize interference.

2. Lymphocytes were identified by combined gating based on CD45 expression and side scatter area (SSC-A), and the P2 gate was established to exclude monocytes and granulocytes and define the lymphocyte population.

3. CD3-expressing cells within the P2 gate were selected to establish the P3 gate, defining CD3⁺ T cells.

4. Within the P3 gate, CD8-expressing cells were selected to establish the P4 gate, defining CD3⁺CD8⁺ T cells, whereas CD4-expressing cells were selected to establish the P5 gate, defining CD3⁺CD4⁺ T cells.

5. Within the P5 gate, cells expressing CD25 but lacking CD127 expression were selected to establish the P6 gate and were defined as CD3⁺CD4⁺CD25⁺CD127⁻ regulatory T cells.

**Fig. 1 Gating strategy for peripheral blood lymphocyte subsets.**
